# Application of a maximal-clique based community detection algorithm to gut microbiome data reveals driver microbes during influenza A virus infection

**DOI:** 10.3389/fmicb.2022.979320

**Published:** 2022-10-20

**Authors:** Anirban Bhar, Laurin Christopher Gierse, Alexander Meene, Haitao Wang, Claudia Karte, Theresa Schwaiger, Charlotte Schröder, Thomas C. Mettenleiter, Tim Urich, Katharina Riedel, Lars Kaderali

**Affiliations:** ^1^Institute of Bioinformatics, University Medicine Greifswald, Greifswald, Germany; ^2^Institute of Microbiology, University of Greifswald, Greifswald, Germany; ^3^Friedrich-Loeffler-Institut, Greifswald-Insel Riems, Greifswald, Germany

**Keywords:** 16S rRNA gene sequencing, microbiome, metaproteome, influenza A virus infection, community detection

## Abstract

Influenza A Virus (IAV) infection followed by bacterial pneumonia often leads to hospitalization and death in individuals from high risk groups. Following infection, IAV triggers the process of viral RNA replication which in turn disrupts healthy gut microbial community, while the gut microbiota plays an instrumental role in protecting the host by evolving colonization resistance. Although the underlying mechanisms of IAV infection have been unraveled, the underlying complex mechanisms evolved by gut microbiota in order to induce host immune response following IAV infection remain evasive. In this work, we developed a novel Maximal-Clique based Community Detection algorithm for Weighted undirected Networks (MCCD-WN) and compared its performance with other existing algorithms using three sets of benchmark networks. Moreover, we applied our algorithm to gut microbiome data derived from fecal samples of both healthy and IAV-infected pigs over a sequence of time-points. The results we obtained from the real-life IAV dataset unveil the role of the microbial families *Ruminococcaceae, Lachnospiraceae, Spirochaetaceae* and *Prevotellaceae* in the gut microbiome of the IAV-infected cohort. Furthermore, the additional integration of metaproteomic data enabled not only the identification of microbial biomarkers, but also the elucidation of their functional roles in protecting the host following IAV infection. Our network analysis reveals a fast recovery of the infected cohort after the second IAV infection and provides insights into crucial roles of *Desulfovibrionaceae* and *Lactobacillaceae* families in combating Influenza A Virus infection. Source code of the community detection algorithm can be downloaded from https://github.com/AniBhar84/MCCD-WN.

## 1. Introduction

Trillions of microorganisms are living inside of multicellular, living organisms (Elgamal et al., 2021). The bacteria in the gut microbiome play an instrumental role in food digestion, regulation of the immune system and protecting the host against infectious disease caused by pathogenic bacteria. The microbial community is formed by interacting microorganisms which act as either predators or symbionts. Symbionts interact with each other in order to gain benefits from each other whereas, predators grapple with each other for the same source of nutrition (Kuntal et al., 2019). Dysbiosis, caused by changes in the gut microbiome, may not only trigger autoimmune diseases such as obesity, hypertension, diabetes, allergic disorders such as food allergy (Lee et al., 2021), but can also lead to the development of colorectal, hepatocellular and breast cancers (Vernocchi et al., 2020).

Influenza A Virus (IAV) is known to cause acute upper respiratory tract infection in animals such as human, pig etc. Following IAV infection, the host immune system plays an instrumental role to counter the infection and prevent serious disease, whereas IAV tries to provoke viral replication by escaping from host's immune surveillance (Chen et al., 2018). Interaction between commensal microbiota and invading viruses often disrupts the healthy gut microbiota community and such disruptions facilitate increasing the transmission of viral infection (Li et al., 2019). In order to protect the host from viral infection, microbiota adopts complex mechanisms such as induction of host immune response (Khan et al., 2021). Previous studies showed the occurrences of co-infections with viral pathogens in a substantial number of patients with a respiratory tract disease (Stefanska et al., 2013; Hoefnagels et al., 2021; Pacheco et al., 2021; Pettigrew et al., 2021). The presence of certain diseases in mammals may lead to an alteration in microbial community and such alteration may have an impact on host-immune response. Although a number of studies have been carried out to unravel the underlying mechanisms of IAV infection (Yildiz et al., 2018; Kaul et al., 2020), the mechanisms through which microbiota evolves the induction of host immune response still remain unknown. Hence, it is necessary to gain a better understanding of the interaction between microbial groups and provide insights into the driver microbial families and their functions that facilitate the induction of the host immune system in order to mitigate IAV infection.

Due to the advancement of next-generation sequencing technology such as 16s ribosomal RNA (rRNA) sequencing, it has become feasible not only to profile hundreds of microorganisms from a single analysis, but also semi-quantify the relative abundance of microbiome members during the progression of infectious diseases. However, although 16s rRNA sequencing has been widely used to mine the microbiome, while it provides information about abundance of species, it gives only very limited information about the function of identified microbial communities (Cortes et al., 2019). This limitation is dealt with the use of shotgun metagenomics. Since, shotgun metagenomics is expensive, proteomics has turned up as an alternative approach. Metaproteomics facilitates the study of all proteins in microbiomes, facilitating an understanding of the functional consequences of the microbiome (Cortes et al., 2019). Hence, the integration of both 16s rRNA sequencing and metaproteomics data may be advantageous to unveil key microbiome families and their role in the progression of infectious diseases.

Given the complexity of the microbial interactions, gut microbiota can be modeled as a network where each node represents an Amplicon Sequence Variant (ASV) and an edge between each pair of nodes represents a predicted association between the corresponding ASVs. In the context of network analysis, community detection algorithms are often used to partition a set of nodes into a number of communities, where nodes belonging to each community are tightly connected with each other. Nodes in each community are supposed to have many within-community edges, but few between-community edges (Yang et al., 2016). Many community detection algorithms have been proposed over the last two decades (Newman, 2004; Derényi et al., 2005; Ahn et al., 2010; Horvath, 2011; Alvarez et al., 2015; Benson et al., 2016; Yang et al., 2016; Lu et al., 2018). These algorithms are broadly categorized into modularity optimization based approaches (Newman, 2006a; Reichardt and Bornholdt, 2006; Blondel et al., 2008), clique based methods (Derényi et al., 2005; Benson et al., 2016; Lu et al., 2018), minimum-cut based methods (Newman, 2004) and hierarchical clustering based approaches (Ahn et al., 2010; Horvath, 2011; Alvarez et al., 2015).

Clique-based community detection methods have achieved augmented attention over the last decades (Derényi et al., 2005; Benson et al., 2016; Lu et al., 2018). One such algorithm is the *k*-clique percolation method (Derényi et al., 2005) which starts with finding all the *k*-cliques in a graph and subsequently merges two *k*-cliques having *k* − 1 edges in common between them. A single giant clique can be produced if the value of *k* is small, whereas a larger value of *k* yields multiple small communities. Hence, the selection of *k* affects the outcome of the algorithm. Another algorithm (Benson et al., 2016) based on network motifs was proposed to detect communities in a given graph. Moreover, this algorithm uses the size of the network motifs to build the co-occurrence count matrix in which each value is set to the number of motifs containing the corresponding pair of nodes. Thus, this algorithm ignores the edge weights and the effects of partitions on the co-occurrence count matrix. Recently (Lu et al., 2018), proposed another clique based community detection algorithm which constructs a clique-conductance matrix accumulating both the size and number of maximal cliques containing a pair of vertices. Although this algorithm overcomes the limitations of the algorithm proposed by Benson et al. (2016), it ignores the edge weights. In addition, most of the existing clique-based algorithms (Derényi et al., 2005; Benson et al., 2016; Lu et al., 2018) use only the local properties of nodes to compute the similarity matrix and ignore the global importance of the nodes. In this paper, we proposed a novel Maximal-Clique based Community Detection algorithm for Weighted Network (MCCD-WN) in order to partition an undirected weighted graph by leveraging the information contained in all the maximal cliques in the given graph and taking both the local and global influence of the nodes into consideration. Moreover, maximal-cliques have biological importance in microbial co-occurrence network. Connectivity between the nodes tends to be higher in infected microbial network compared to healthy microbial network due to colonization activity during infection. Such variability in the node connectivity can be observed in maximal-clique structure.

The identified communities can further be used to detect different types hubs such as kinless, provincial and connector hubs. Theoretically the connector and provincial hubs are defined as the nodes which are highly connected between the modules and within a module, respectively. The kinless hubs are highly connected not only between the modules, but also within a module and hence, the kinless hubs are functionally more important to conciliate stabilization in the functional networks in the gut microbiome (Mangangcha et al., 2020; Shi et al., 2020). In this work, we applied our MCCD-WN algorithm to a time-series 16s rRNA sequencing dataset containing the abundance of ASVs in healthy and IAV infected cohorts in order to (i) identify driver ASVs and their corresponding microbial families at each time-point, (ii) find out different types of hub ASVs in the co-abundance network and their corresponding families at each time-point and (iii) provide insights into the functionalities of the microbiome families of the identified driver and hub ASVs at each time-point for both the healthy and infected states by integrating a metaproteome data.

## 2. Materials and methods

### 2.1. Materials

#### 2.1.1. Artificial datasets

In order to evaluate the performance of the proposed community detection algorithm MCCD-WN, we used the LFR benchmark (Lancichinetti et al., 2008; Lancichinetti and Fortunato, 2009), which is a generalization of Girvan-Newman benchmark (Girvan and Newman, 2002) and assumes a power-law degree distribution of the nodes, similar to biological networks (Jing et al., 2021). The LFR benchmark uses a parameter called mixing parameter (μ_*w*_) which is delineated as the ratio of the external degree of a node to the total degree of the node. Hence, a higher value of μ_*w*_ disrupts the community structure as each node will have a higher external degree than within-community degree. In this work, we generated three different sets of networks, *AN*_1_, *AN*_2_, and *AN*_3_ using the LFR benchmark with 500, 3000 and 5000 nodes, respectively. Each of these three sets of networks contains a set of disjoint communities. For *AN*_1_ and *AN*_3_, we set the parameters *k* (average degree) to 25, *beta* (exponent for the weight distribution) to 1.1, *minc* (minimum for the community sizes) to 10, and *maxc* (maximum for the community sizes) to 50. In case of *AN*_2_, we set the parameters *k* (average degree) to 15, *beta* (exponent for the weight distribution) to 1.1, *minc* (minimum for the community sizes) to 20, and *maxc* (maximum for the community sizes) to 50. The values of *maxk* (maximum degree) for *AN*_1_, *AN*_2_ and *AN*_3_ benchmark networks are set to 50, 300, and 500, respectively. The value of the mixing parameter for the edge weights (μ_*w*_) varies from 0.1 to 0.8 for each set of the networks.

#### 2.1.2. Real-life datasets

The real data we use is from an influenza infection experiment carried out in pigs at the Department of Experimental Animal Facilities and Biorisk Management of the Friedrich Loeffler Institute within the H1N1pdm09 animal experiment (Schwaiger et al., 2019; Gierse et al., 2020, 2021). H1N1 infection was carried out in 19 pigs, three additional animals were used as controls. Fecal samples of all animals in the healthy cohort were used subsequently to generate 16s rRNA gene sequences, while samples of 19, 16, 12, and eight animals in the infected cohort were taken at days 0, 7, 21, and 25, respectively. Sequences obtained from 16s rRNA sequencing experiment of the infected cohort are available at European Nucleotide Archive (ENA) with project number PRJEB42450 and accession number ERP126308 whereas, the sequences for the healthy cohort can be downloaded from European Nucleotide Archive (ENA) using project number PRJEB39963 and accession number ERP123542. Fecal samples of all pigs from the healthy cohort and three pigs from the infected cohort were used for metaproteomic analysis. The mass spectrometry proteomics data can be obtained from ProteomeXchange Consortium using the dataset identifier PXD020775. Abundances of the microbial proteins measured across the same set of time-points i.e., days 0, 7, 21, and 25, were used in this work. Days 0 and 21 are reported to be the days of first and second IAV infection, respectively. The purpose of the second infection was to trigger the host immune response and explicate the crosstalk between host immune system and gut microbiome. Moreover, it is reported that the presence of influenza A virus matrix protein was observed after the first infection, whereas it was not detected after the second infection, implying a fast recovery of the infected animals (Gierse et al., 2021).

### 2.2. Methods

**Figure 1:**
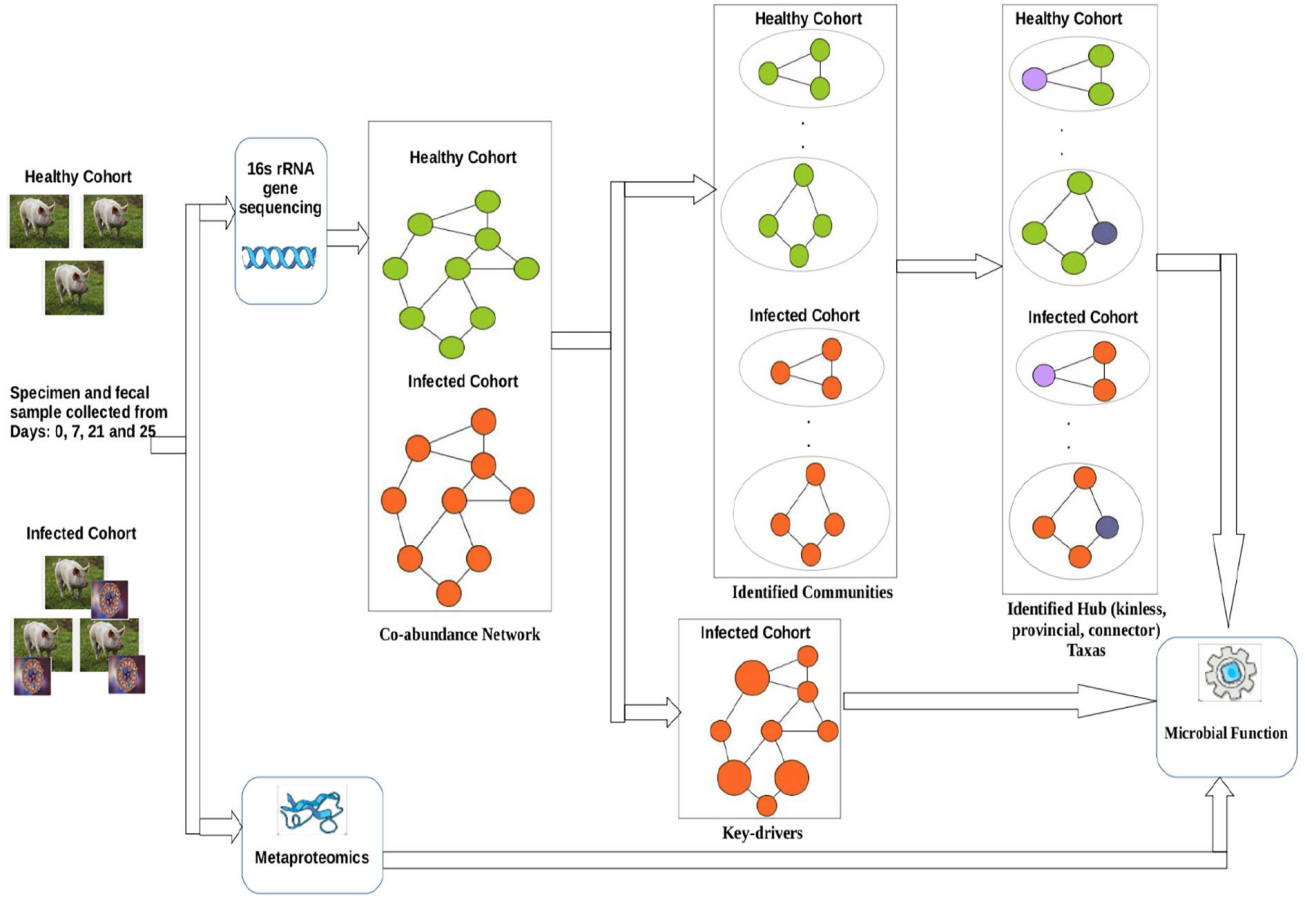
Workflow used in this work.

Figure 1 shows a schematic diagram of the overall workflow used in this work. Initially, co-abundance networks were built for both the healthy and infected cohorts. Subsequently, the co-abundance networks were analyzed to extract the driver ASVs. In addition, the co-abundance networks were used to find communities which were thereafter used to provide insights into the role of microbial families in subduing pathogen colonization and identify ASVs acting as different types of hubs. Finally, we leveraged the metaproteomics data in order to provide insights into functional role of microbial families of the identified driver and hub ASVs.

#### 2.2.1. Co-abundance network construction

In order to build a reliable co-abundance network, amplicon sequence variants (ASVs) present in at least 30% of all the samples and having an average relative abundance value > 0.01% were used. SparCC, a method designed to estimate the correlation values for compositional data (Friedman and Alm, 2012), was then used to compute the correlation coefficient between each pair of ASVs. The significance of the correlation coefficients was established using randomly permuted data and a *p*-value cutoff < 0.05. In this work, we built one network at each time-point for both the healthy and infected cohorts.

#### 2.2.2. Identification of key-drivers

**Figure 2:**
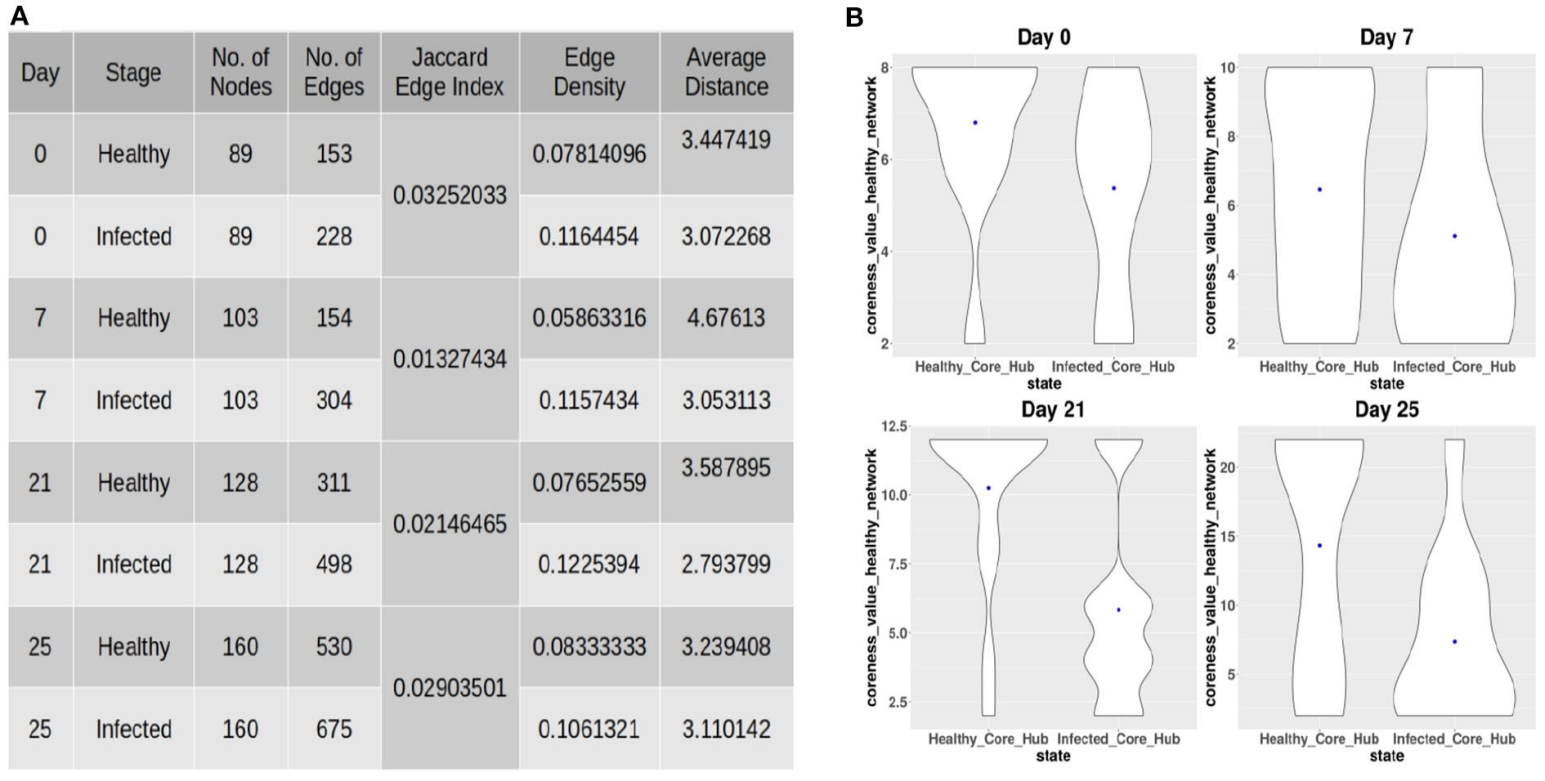
**(A)** Topological properties of healthy and infected co-abundance networks at each of the time-points. **(B)** Coreness values of the ASVs belonging to the infected-core-hub community in the control network and the ASVs belonging to the control-core-hub community.

After constructing the co-abundance network at each time-point, we considered ASVs present in both the healthy and infected networks in order to obtain a comparable set of nodes for performing a comparative analysis on both the networks. The properties of the co-abundance networks were computed using the R package *igraph* (Csardi and Nepusz, 2006). The topological properties of each of the co-abundance networks are shown in Figure 2A. For each of the time-points, a lower Jaccard Edge Index (JEI) score between the healthy and infected co-abundance networks implies the occurrence of rewiring. Moreover, it is of interest to observe that the infected network has a higher edge density and lower average distance as compared to the healthy network at each time-point. This tendency might be an indication of colonization activity in the infection setting (Kuntal et al., 2019). In order to identify driver ASVs at each time-point, we used the neighborhood shift (netshift) score (Kuntal et al., 2019). The netshift score of a node *i* is computed as


(1) $$\begin{array}{l} nesh\left(i\right)=1-\left(T1-\left(T2+T3\right)\right), \end{array}$$

where


(2) $$\begin{array}{l} T1=\frac{{\left[\Lambda_{i}\right]}^{H}\cap {\left[\Lambda_{i}\right]}^{I}}{{\left[\Lambda_{i}\right]}^{H}\cup {\left[\Lambda_{i}\right]}^{I}}, \end{array}$$

(3) $$\begin{array}{l} T2=\frac{{\left[\Lambda_{i}\right]}^{I}-{\left[\Lambda_{i}\right]}^{H}}{max\;degree\;in\;I}, \end{array}$$

(4) $$\begin{array}{l} T3=\frac{{\left[\Lambda_{i}\right]}^{I}-{\left[\Lambda_{i}\right]}^{H}}{{\left[\Lambda_{i}\right]}^{H}\cup {\left[\Lambda_{i}\right]}^{I}}, \end{array}$$

and ${\left[\Lambda_{i}\right]}^{I}$ (or ${\left[\Lambda_{i}\right]}^{H}$) represents the set of direct neighbors of node *i* in the infected (or healthy) network. A node having a higher *nesh* score is considered to have a higher neighborhood shift in the infected network over a healthy network. Since the netshift score does not account for the edge weights between a node and its interacting partners, we used the betweenness centrality score and a Shannon entropy based node importance metric to estimate the importance of each node in the healthy and infected networks. The betweenness centrality score (Kuntal et al., 2019) of the *i*th node in the healthy (or infected) network is normalized as


(5) $$\begin{array}{l} BW_{scaled}\left(i\right)=\frac{BW\left(i\right)-BW_{min}}{BW_{max}-BW_{min}}, \end{array}$$

where *BW*(*i*) represents the betweenness value of node *i* in the healthy (or infected) network, and *BW*_*min*_ and *BW*_*max*_ denote the minimum and maximum values of all betweenness centrality scores in the healthy (or infected) network, respectively. Subsequently, the relative importance of the *i*th node is computed using equation-6


(6) $$\begin{array}{l} \Delta BW\left(i\right)=\left(BW_{{scaled}_{I}}\left(i\right)-BW_{{scaled}_{H}}\left(i\right)\right), \end{array}$$

where *BW*_*scaled*_*H*__(*i*) and *BW*_*scaled*_*I*__(*i*) denote the normalized betweenness centrality scores of node *i* in the healthy and infected networks, respectively. A node having a positive Δ*BW* value has more importance in the infected network than in the healthy network. In addition to the betweenness centrality score, we used a Shannon entropy based metric to estimate the node importance based on its strength in the healthy and infected network. The Shannon entropy of a node *i* (Li et al., 2015) can be computed as


(7) $$\begin{array}{l} ℍ\left(i\right)=-\overset{n}{\underset{j=1}{\sum }}p_{ij}log\left(p_{ij}\right), \end{array}$$

where $p_{ij}=\frac{w_{ij}}{\overset{n}{\underset{j=1}{\sum }}w_{ij}}$; *w*_*ij*_ denotes the weight between nodes *i* and *j*. Subsequently, the entropy of a network is computed as


(8) $$\begin{array}{l} ℍ\left(V\right)=-\overset{n}{\underset{i=1}{\sum }}\overset{n}{\underset{j=1}{\sum }}p_{ij}log\left(p_{ij}\right), \end{array}$$

where *V* represents the set of *n* number of nodes in the network. In order to compute the importance of a node *i* in the network, we removed the *i*th node along with its edges from the network and then computed the entropy (ℍ(*V*−*i*)) of the resultant graph (Omar and Plapper, 2020). Subsequently, the normalized node importance score is computed using equation 9 so that ℍ(*V*) is always scaled to 1,


(9) $$imp_{scaled}(i)=\frac{ℍ(V-i)-min_{\forall i\in V}(ℍ(V-i))}{ℍ(V)-min_{\forall i\in V}(ℍ(V-i)}.$$

Finally, the relative importance of the *i*th node based on the Shannon entropy based metric is computed as


(10) $$\begin{array}{l} \Delta imp\left(i\right)=\left(imp_{{scaled}_{I}}\left(i\right)-imp_{{scaled}_{H}}\left(i\right)\right), \end{array}$$

where *imp*_*scaled*_*I*__(*i*) and *imp*_*scaled*_*H*__(*i*) denote the importance of the *i*th node in the infected and healthy networks, respectively. An ASV *i* is considered to be a driver ASV if it has a higher neighborhood-shift score and positive Δ*BW*(*i*) and Δ*imp*(*i*) values.

#### 2.2.3. Identification of modules of bacterial ASVs

To extract modules from the co-abundance network, we applied a novel community detection algorithm MCCD-WN which leverages all the maximal cliques of a given network. An undirected weighted graph is defined as *G* = {*V, E, W*}, where *V* corresponds to a set of vertices, *E* corresponds to a set of edges and *W* is a symmetric weight matrix of size ||*V* × *V*|| where each element *W*[*i, j*] represents the weight between each pair of connected vertices *i* and *j*. In graph theory, a clique is defined as a subgraph where every pair of nodes is connected with each other. A maximal clique is a clique that can not be expanded by adding any other vertex which is not a part of it. A *k*-clique is a clique having *k* number of vertices. In this work, we leveraged all the maximal *k*-cliques to find a set of modules in a given network by ensuring that there are very few cliques having lower density between different modules, whereas the number of cliques having higher density within the same module is relatively high. To achieve our goal, we first constructed a similarity matrix by utilizing both the global importance and local information of the nodes. In order to compute the global importance of the *i*th node, we used the PageRank algorithm (Page et al., 1999). PageRank method aims at ranking a set of web pages by assigning a weight based on the behavior of a random surfer which is similar to a Markov chain process. The estimated weight of a node represents the probability that a random surfer visits a web page either by following a hyperlink or directly by entering the address of the page in the web browser. Thus a pagerank score captures the influence of a node on every other vertex in a network and can be used as a global importance score of a node in a given network. The pagerank score is computed by equation 11


(11) $$\begin{array}{l} imp\text{\_}global_{i}=\beta \underset{j\in \Lambda_{i}}{\sum }\frac{imp\text{\_}global_{j}}{deg_{j}}+\frac{1-\beta }{N}, \end{array}$$

where Λ_*i*_ denotes the direct neighbors of the *i*the node, *deg*_*j*_ corresponds to the degree of node *j* and *N* denotes the total number of vertices in the given graph. In the PageRank algorithm, β ∈ {0, 1} is used to denote the probability that a random surfer will continue to follow the hyperlink structure and is usually set to 0.85. Subsequently, we used Equation (12) to compute each element of the first structural similarity matrix *Sim*_1, based on the global importance values of the nodes,


(12) $$\begin{array}{l} Sim\text{\_}1\left[i,j\right]=\frac{\sum_{x\in \left(\Lambda_{i}\cap \Lambda_{j}\right)}imp\text{\_}global_{x}}{\sqrt{\sum_{x\in \Lambda_{i}}imp\text{\_}global_{x}}\sqrt{\sum_{x\in \Lambda_{j}}imp\text{\_}global_{x}}}. \end{array}$$

The weight matrix *W* is used as the second similarity matrix *Sim*_2 as denoted by Equation (13):


(13) $$\begin{array}{l} Sim\text{\_}2\left[i,j\right]=W\left[i,j\right]. \end{array}$$

Finally, Equation (14) was used to compute the final similarity matrix,


(14) $$\begin{array}{l} Sim\left[i,j\right]=\left(\alpha *Sim\text{\_}1\left[i,j\right]\right)+\left(\left(1-\alpha \right)*Sim\text{\_}2\left[i,j\right]\right), \end{array}$$

where the value of α is set experimentally. In the context of spectral clustering, the problem of optimizing the conductance function is considered as computationally intractable and can be converted into a relaxed, tractable eigenvector problem in order to obtain an approximate solutions. In the context of an undirected weighted graph partitioning problem, the conductance function is given by


(15) $$\begin{array}{l} \phi \left(C_{1},C_{2},..,C_{K}\right)=\frac{cut\left(C_{i},\bar{C_{i}}\right)}{volume\left(C_{i}\right)}, \end{array}$$

where {*C*_1_, *C*_2_, .., *C*_*K*_} are the set of communities such that (*C*_*i*_ ∩ *C*_*j*_) = ∅, ∀(*i, j*)_*i*≠*j*_ ∈ {1, 2, .., *K*} and ⋃_*i*_*C*_*i*_ = *V*. In spectral clustering, we aim at minimizing the ratio ϕ(*C*_1_, *C*_2_, .., *C*_*K*_). Moreover, the normalized cut ensures maximization of the intra-cluster similarity as long as the *volume*(*C*) is maximized and the cut with the remaining vertices is minimized.

Now, we define the clique conductance function based on the information obtained from all maximal *k*-cliques in order to partition the given undirected weighted graph. The weight matrix for a clique-induced graph is computed using Equation (16),


(16) $$\begin{array}{l} W_{clq}\left[i,j\right]=\underset{l\in Clq_{k}}{\sum }\frac{\sum_{\left(i,j\right)\in l}Sim\left[i,j\right]}{||l||*\left(||l||-1\right)}, \end{array}$$

where *Clq*_*k*_ denotes the set of all maximal *k*-cliques, ||*l*|| is the size of *l*th maximal clique and *W*_*clq*_[*i, j*] represents how tightly two vertices *i* and *j* are connected in the *l*th maximal *k*-clique.

In the context of a clique conductance function optimization problem, $cut\left(C,\bar{C}\right)=\underset{i\in C,j\in \bar{C}}{\sum }W_{clq}\left[i,j\right]$ and $volume\left(C\right)=\underset{i\in C,j\in V}{\sum }W_{clq}\left[i,j\right]$. Although the identification of all maximal cliques is known to be an NP-hard problem, retrieval of all maximal cliques is not problematic because the co-abundance network of bacterial ASVs is sparse. Algorithm 1 demonstrates the steps of our method to partition a weighted undirected graph. For a binary clustering problem, we divided the nodes into two groups based on the sign of values in the eigenvector corresponding to the second smallest eigenvalue of the normalized laplacian matrix *L*_*clq*_ of the clique-induced graph. In order to find more than two clusters, we have used the *K*-median algorithm as it is less sensitive to outliers. The problem of computing cluster centroids is converted into a *L*_1_-median computing problem and solved using the algorithm proposed by Vardi and Zhang (2000). The *L*_1_-median computing algorithm proposed by Vardi et al., an improved version of the Weiszfeld algorithm (Weiszfeld and Plastria, 2009), is capable of dealing with the situation where the median is found to be one of the data points in the cluster. In order to obtain better performance and reproducible clusters, we used Algorithm 2 which is similar to the *K*-means++ algorithm (Arthur and Vassilvitskii, 2007) for initial centroids selection. Algorithm 2 starts with finding a seed node as the one having the lowest betweenness centrality score. The intuition behind the selection of the seed node is that the seed node is unlikely to act as a bridge node between different communities.

**Algorithm 1:** Proposed weighted graph partitioning algorithm.

**Require:** Undirected weighted graph *G* = {*V, E, W*}, where *W* ∈ ℝ^*n*×*n*^ and *K*, the number of communities.
**Ensure:** A set of disjoint communities {*C*_1_, *C*_2_, .., *C*_*K*_}
**Step I**. Extract all maximal cliques using the algorithm proposed by Eppstein et al. (2010)
**Step II**. Compute the weight matrix *W*_*clq*_, for the clique induced graph using equation 16.
**Step III**. Compute the degree matrix *D*_*clq*_ from *W*_*clq*_ and normalized laplacian matrix *L*_*clq*_ using $D_{clq}^{-\frac{1}{2}}\left(D_{clq}-W_{clq}\right)D_{clq}^{-\frac{1}{2}}$.
**if** *K* = 2 **then**
**Step IVa**. Compute the second eigenvector $E_{clq}\in {\mathbb{R}}^{n}$ of *L*_*clq*_
**Step IVb**. Assign *i*th object to community *C*_1_ if the *i*th element of *E*_*clq*_ is greater than 0; otherwise, assign *i*th object to community *C*_2_.
**else**
**Step Va**. Compute the first *K* eigenvectors $M_{clq}\in {\mathbb{R}}^{n\times K}$ of *L*_*clq*_
**Step Vb**. Compute a matrix $T_{clq}\in {\mathbb{R}}^{n\times K}$ by normalizing the rows of matrix *M*_*clq*_ to norm 1 by setting $T_{clq}\left[i,j\right]=\frac{M_{clq}\left[i,j\right]}{{\left(\overset{K}{\underset{k=1}{\sum }}M_{clq}{\left[i,k\right]}^{2}\right)}^{\frac{1}{2}}}$
**Step Vc**. Select *K* number of initial centroids using Algorithm 2.
**Step Vd**. Apply *K*-median clustering algorithm to *T*_*clq*_ to get *K* number of disjoint communities {*C*_1_, *C*_2_, .., *C*_*K*_}
**end if**

**Algorithm 2:** Initial centroids selection algorithm.

**Require:** Undirected weighted graph *G* = {*V, E, W*}, where *W* ∈ ℝ^*n*×*n*^, normalized matrix $T_{clq}\in {\mathbb{R}}^{n\times K}$ and *K*, the number of communities.
**Ensure:** *K* number of initial centroids.
**Step I**. *S* ← ∅
**Step II**. Compute the betweenness centrality score of each vertex in the graph *G* and select the vertex (*p*) having the lowest betweenness centrality score as the initial centroid.
**Step III**. *S* ← (*S* ∪ *p*)
**while** ||*S*|| < *K* **do**
**Step IVa**. For each data point *q* ∉ *S*, compute the distance (*dist*(*q*)) between *q* and the nearest point in *S*.
**Step IVb**. Select a new point *q* ∉ *S* having the highest probability value $\frac{dist{\left(q\right)}^{2}}{\underset{q\notin S}{\sum }dist{\left(q\right)}^{2}}$, as the new center and add it to *S* (*S* ← (*S* ∪ *q*))
**end while**

#### 2.2.4. Estimating the number of communities

**Figure 3:**
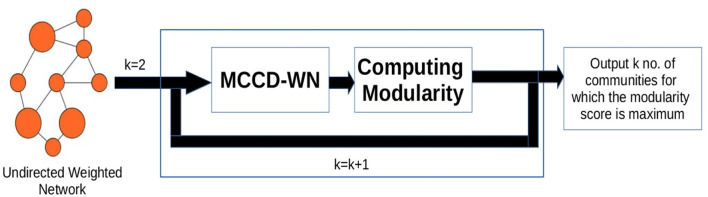
Workflow for estimating the number of communities. *k* denotes the number of communities.

In order to estimate the number of communities, we used an iterative approach shown in Figure 3. We applied our algorithm to extract different number (*K*) of communities and computed the modularity score (*Q*) (Newman, 2006b) for every value of *K* using Equation (17),


(17) $$\begin{array}{l} Q=\frac{1}{2m}\underset{i,j\in V}{\sum }\left(W\left[i,j\right]-\frac{deg_{i}deg_{j}}{2m}\right)\delta \left(C_{i},C_{j}\right), \end{array}$$

where *deg*_*i*_ and *deg*_*j*_ denote the degree of nodes *i* and *j*. δ(*C*_*i*_, *C*_*j*_) is set to 1 if both the nodes belong to the same community, otherwise δ(*C*_*i*_, *C*_*j*_) is set to 0. *W*[*i, j*] represents the weight of the edge between nodes *i* and *j*. *m* is the sum of all the edge weights in the network. Finally, the number of communities is set to the value of *K* which results in the maximum modularity (*Q*) score.

#### 2.2.5. Identification of ASVs preventing pathogen colonization

In order to provide insights into the commensal bacteria playing a crucial role in conquering pathogen colonization, we identified the core-hub community in both the healthy and infected networks as the one having the highest coreness value. Subsequently, we computed the coreness values of the ASVs belonging to the infected-core-hub community in the control network and compared with the coreness values of the ASVs belonging to the healthy-core-hub community. Figure 2B shows that the ASVs in the infected-core-hub community have lower coreness values in the healthy network as compared to the healthy-core-hub community members at each time-point. This phenomenon may imply the prevention of pathogen colonization by the corresponding microbial families of the commensal bacterial ASVs.

#### 2.2.6. Identification of hub ASVs

We computed the participation coefficient score (Hall et al., 2019) for each node using Equation (18),


(18) $$\begin{array}{l} PC_{i}=1-\underset{k\in K}{\sum }\frac{deg_{i}\left(k\right)}{deg_{i}}, \end{array}$$

where *K* denotes the number of communities, *deg*_*i*_(*k*) denotes the degree of node *i* within community *k*. A higher value of *PC*_*i*_ represents that the node *i* has higher inter-module connections, relative to intra-module connections. In addition to the participation coefficient, we calculated the within-community *z*-score (Hall et al., 2019).


(19) $$\begin{array}{l} z_{i}=\frac{deg_{i}\left(C_{i}\right)-\bar{deg}\left(C_{i}\right)}{\sigma^{deg\left(C_{i}\right)}}, \end{array}$$

where *C*_*i*_ is the community containing node *i*, *deg*_*i*_(*C*_*i*_) denotes the intra-module degree of node *i*, $\bar{deg}\left(C_{i}\right)$ and $\sigma^{deg\left(C_{i}\right)}$ stand for the mean and standard deviation of intra-module degrees of the nodes belonging to community *C*_*i*_, respectively. Finally, the nodes having a *z*_*i*_ score greater than 0 are considered to be hubs. Furthermore, a hub node having a *PC*_*i*_ score ≥ 0.75 is defined as a kinless hub, a hub node having a *PC*_*i*_ score ≥ 0.30 and < 0.75 is considered as a connector hub and a hub node having a *PC*_*i*_ score < 0.30 is defined as a provincial hub.

#### 2.2.7. Prediction of microbial biomarkers and their functions associated with healthy and infected cohorts

Since very limited information about the function of microbial community is provided by 16s rRNA sequencing experiment, we leveraged a metaproteome data obtained from the same healthy and infected cohorts for unveiling the functional potential of microbiome family and the microbial proteins during IAV infection. In order to achieve this, we applied Linear discriminant analysis Effect Size (LEfSe) to the relative abundance of functional categories of the protein groups belonging to the microbial families identified as hubs, key-drivers and members of the infected core-hub community at each time-point (Segata et al., 2011). Functional categories having a *LDA* > 2.0 and *p value* < 0.05 are considered to be the ones which explain the difference between the healthy and infected classes. Finally, the LEfSe analysis was carried out using the relative abundance of protein groups belonging to the significantly associated functional categories in order to identify the microbial proteins which differentiate the healthy and infected classes.

## 3. Results

### 3.1. Results on the LFR benchmark datasets

**Figure 4:**
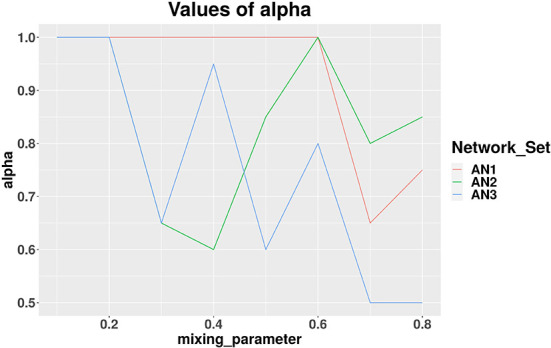
Different values of the parameter α for three sets of LFR benchmark networks *AN*_1_, *AN*_2_, and *AN*_3_.

**Figure 5:**
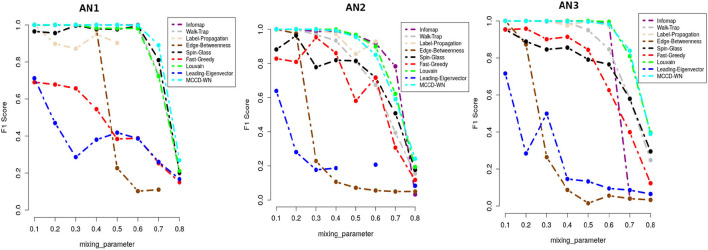
Performance comparison in terms of F1-score.

We applied our MCCD-WN algorithm to three sets of LFR benchmark networks. Although the number of communities can be provided by the user, we estimated the number of communities along with the parameter α by optimizing the modularity score (*Q*). We initialized the value of α to 1 and kept reducing its value by 0.05 in every iteration. Figure 4 shows the changes in the values of α for different values of the mixing parameter (μ_*w*_), in case of each set of LFR benchmark networks. It is of interest to observe that in order to achieve the best performance of the MCCD-WN algorithm, the global node importance score is playing a crucial role for a relatively higher value of the mixing parameter μ_*w*_. In order to evaluate the performance of the proposed MCCD-WN algorithm and compare its performance with the other existing algorithms, we have used the *F*_1_ score (Laarhoven and Marchiori, 2016) as an evaluation metric, which can be defined by equation 20,


(20) $$\begin{array}{l} F_{1}\left(C,C^{*}\right)=2*\frac{\left|C\cap C^{*}\right|}{\left|C|+|C^{*}\right|}, \end{array}$$

where *C* and C^*^ indicate the ground truth community and community produced by the community detection algorithm. The *F*_1_ score denotes how well a community detection algorithm finds the ground-truth communities. *F*_1_ ranges from 0 to 1, where a higher value indicates a better performance of the algorithm. Figure 5 demonstrates that the proposed algorithm obtained the highest *F*_1_ score in case of the benchmark datasets *AN*_1_ (μ_*w*_ = 0.7 and 0.8), *AN*_2_ (μ_*w*_ = 0.8) and *AN*_3_ (μ_*w*_ = 0.7) whereas, it produces very similar *F*_1_ scores as the other best performing algorithms for the remaining LFR benchmark networks.

**Figure 6:**
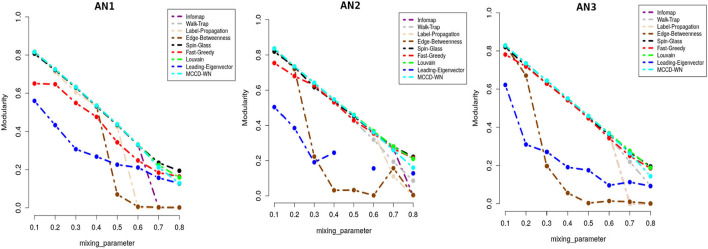
Performance comparison in terms of modularity score.

In addition to *F*_1_ score, we also used Modularity score (*Q*) as delineated in equation 17 for evaluating the performance of the proposed algorithm. Modularity score ranges from –1 to +1 and a higher modularity score indicates a better structure of the communities found in the given network. Figure 6 shows that the proposed algorithm achieved either equal or very close modularity scores to the ones obtained by the best performing algorithms for all the benchmark networks.

**Figure 7:**
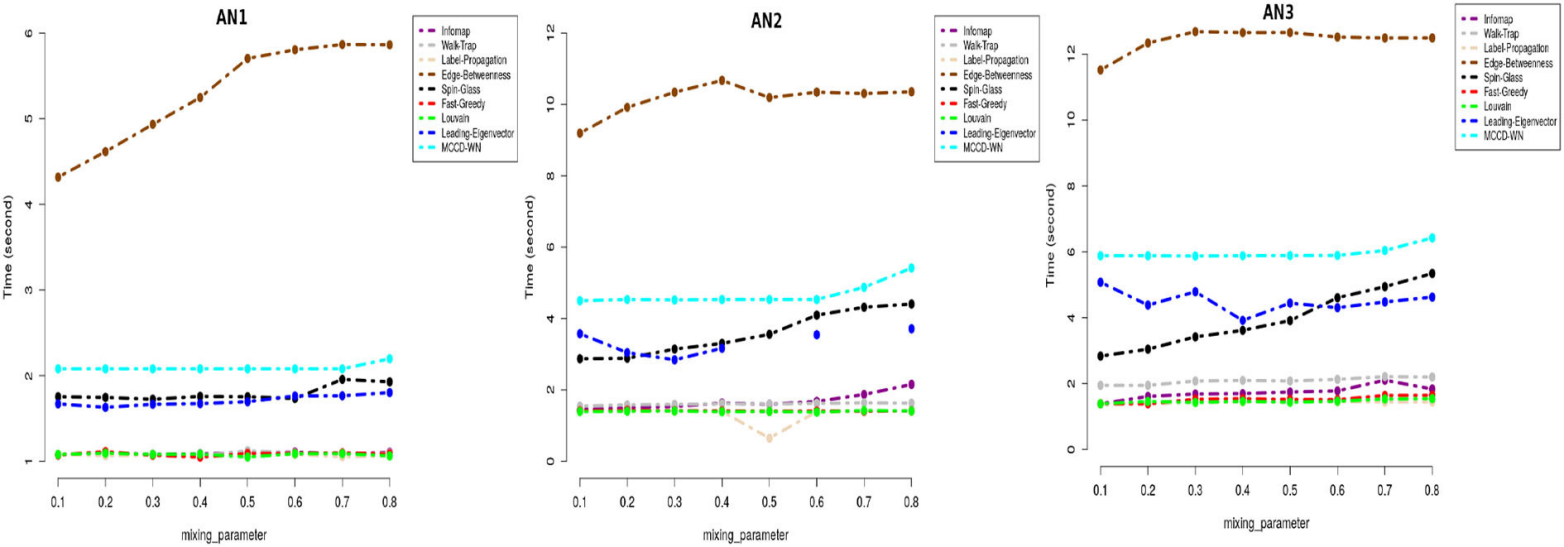
Performance comparison in terms of running time (in seconds) on a log-linear scale.

Moreover we carried out a study on the computing time for each of the benchmark networks. From Figure 7, it can be well seen that the proposed algorithm has reasonable computation speeds on each of the benchmark networks.

#### 3.1.1. Similarity between real networks and LFR benchmark networks

In order to compare the real co-abundance networks obtained from Section 2.2.1 with the LFR benchmark networks, we used *AN*_1_ *in silico* networks. Supplementary Figures 1, 2 compare *AN*_1_ benchmark networks with the co-abundance networks in terms of the number of nodes, number of edges, edge density value and clustering coefficient. Moreover, we estimated the quality of the community structures present in the real networks by computing the absolute difference between the clustering coefficients of the real networks with that of each benchmark network. From Supplementary Figures 3, 4 it can be well seen that the real networks are comparable to the benchmark networks generated using a mixing parameter (μ_*w*_) value less than or equal to 0.5.

### 3.2. Results on real-life data

#### 3.2.1. Reproducibility testing of the proposed workflow

In order to test the reproducibility of the proposed workflow, we computed the size of intersection between the microbial families reported to be instrumental in Borey et al. (2021) and Gierse et al. (2021) and the ones found by applying our workflow to these two datasets. The sequences of the first dataset (Borey et al., 2021) were downloaded from the NCBI Sequence Read Archive using accession number PRJNA647267 and processed using SHAMAN tool (Volant et al., 2020) in order to obtain the OTU abundance matrix and annotation table. In case of the second experiment (Gierse et al., 2021), We used the sequence data obtained from the fecal samples of healthy and infected cohorts. Supplementary Figures 5A,B show that we achieved descent Jaccard similarity coefficient scores 0.70 and 0.66 for these two datasets, respectively.

#### 3.2.2. Infected networks indicate a faster recovery after the second infection

**Figure 8:**
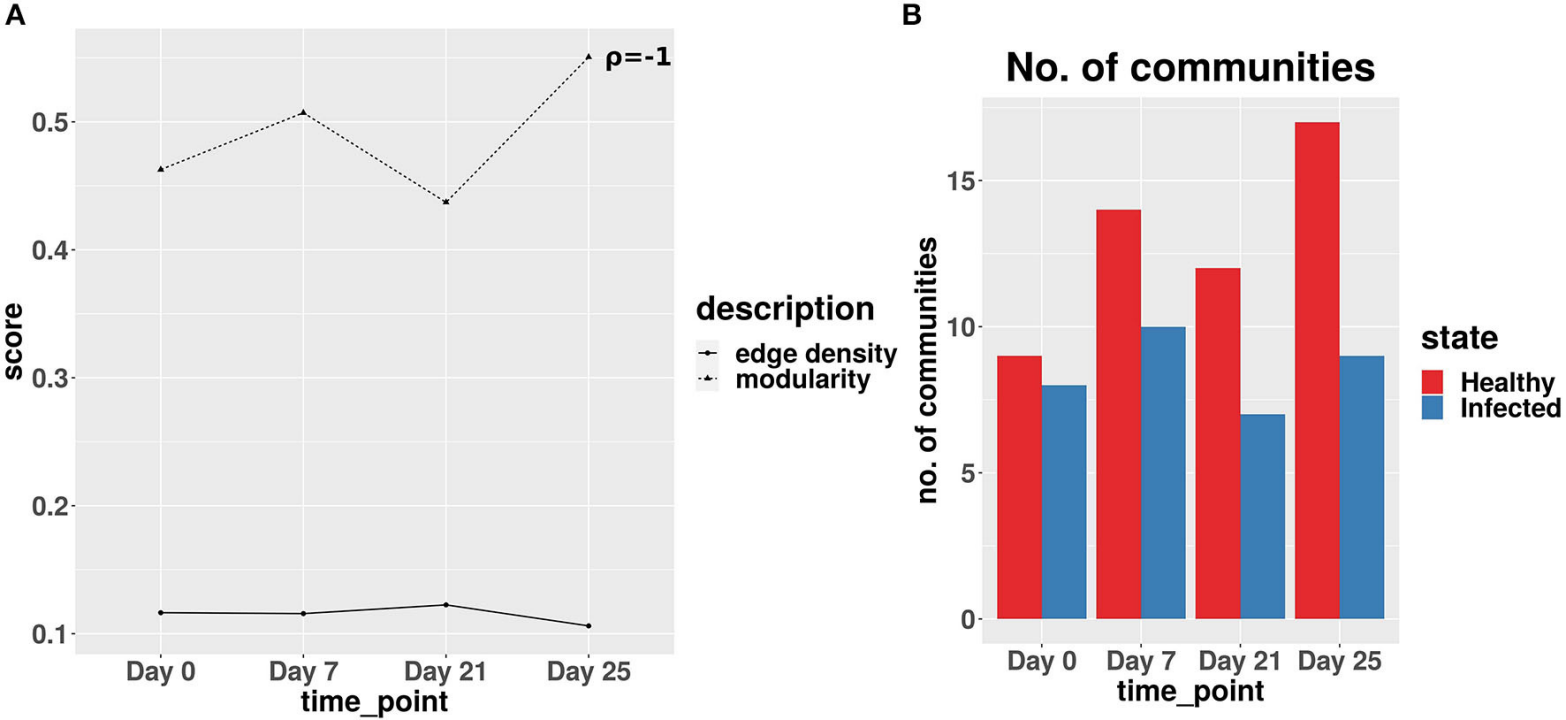
**(A)** Average edge density and modularity scores of the co-abundance networks for infected cohort. ρ denotes the Spearman correlation coefficient between average edge density and modularity scores across all time-points. **(B)** Number of communities obtained for real-life dataset.

The network analysis of gut microbiome data of IAV infected pigs revealed a strong negative correlation between the edge density values and modularity scores of the co-abundance network of infected cohort across all the time-points. Figure 8A exhibits the lowest modularity score and highest edge density value at day 21. This phenomenon indicates the maximum disruption of microbial communities and highest rise in the colonization activity triggered by the second IAV infection, whereas at day 25, a relatively lower edge density value and higher modularity score indicate a reduction in both the colonization activity and disruption in microbial communities, respectively (Baldassano and Bassett, 2016). In Figure 8B, we demonstrated the number of communities found in ASV co-abundance networks for the healthy and infected cohorts at each time-point.

#### 3.2.3. Microbial families for the ASVs identified as key-driver and members of infected-core-hub communities

**Figure 9:**
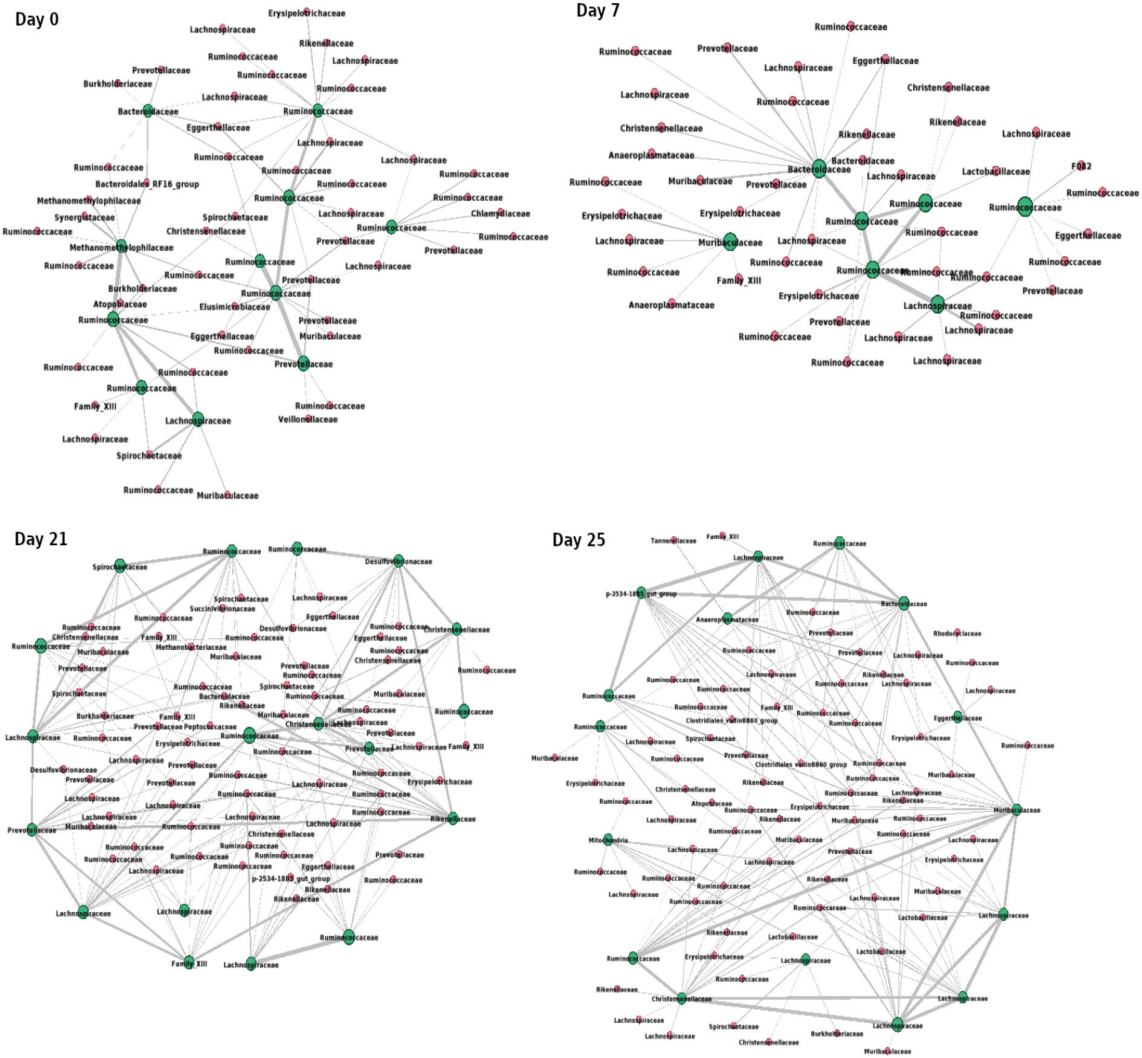
Network consisting of driver ASVs and their interaction partners at each of the time-points. Green nodes represent the names of families of corresponding driver ASVs. Thickness of edges is proportional to the corresponding correlation coefficient value. The network is drawn using Gephi tool (Bastian et al., 2009).

**Figure 10:**
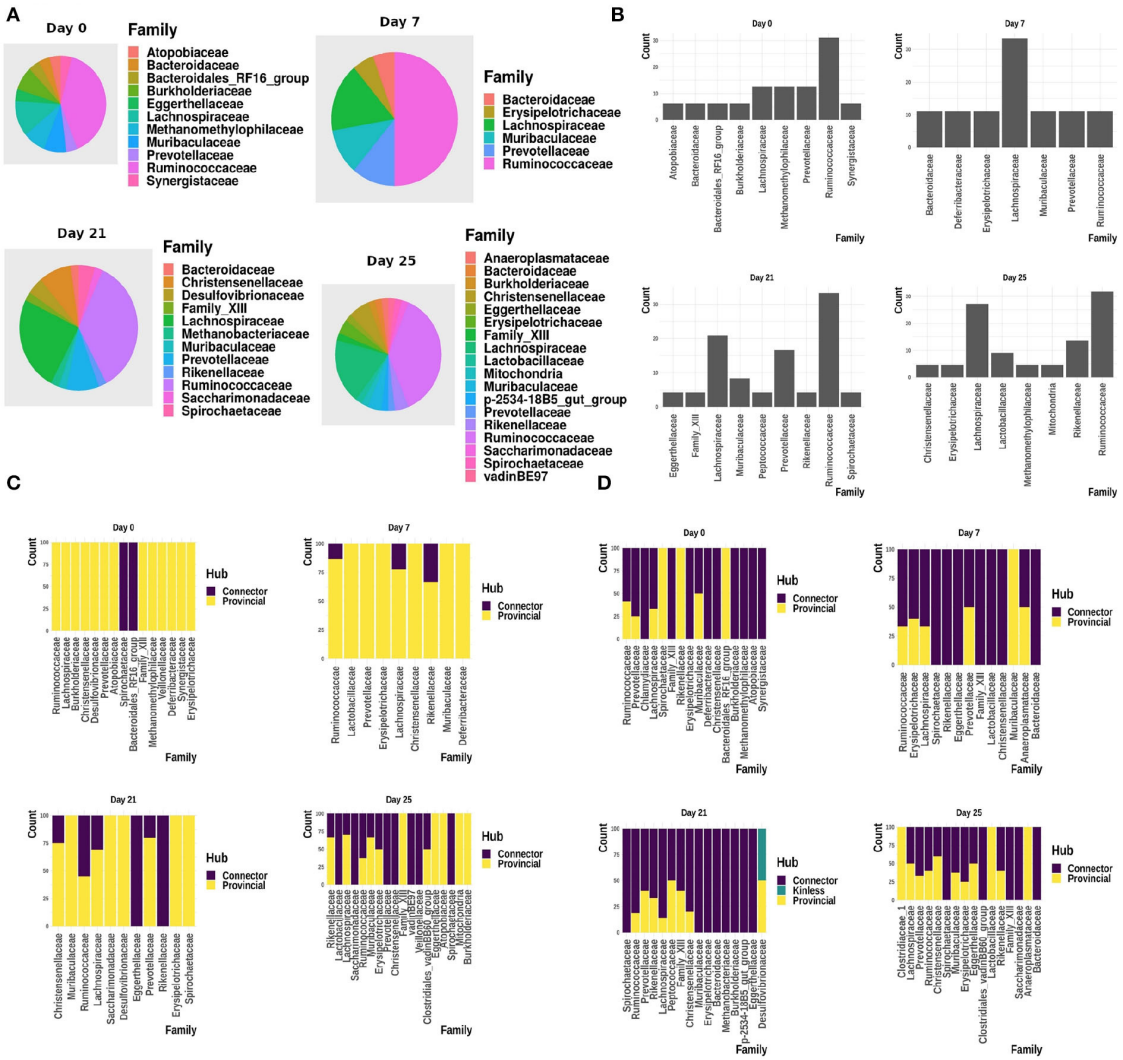
**(A)** Relative proportion of driver ASVs assigned to the corresponding microbiome families at each of the time-points. **(B)** Relative proportion of ASVs belonging to the infected-core-hub communities, assigned to the corresponding microbiome families at each of the time-points. **(C)** Relative proportion of ASVs identified as hubs in healthy co-abundance networks. **(D)** Relative proportion of ASVs identified as hubs in infected co-abundance networks.

Figure 9 shows the interactions between the microbial families of ASVs found to undergo rewiring in the infected co-abundance network and their interaction partners at each of the time-points. In Figure 10A, we can see that at each time-point the majority of the driver ASVs belong to the family *Ruminococcaceae*. Figure 10B shows that the majority of the ASVs in the identified infected-core-hub communities belong to the family *Ruminococcaceae* at Day 0, 21, and 25, whereas at Day 7 most of such ASVs come from the family *Lachnospiraceae*. The microbial families of the ASVs belonging to the infected-core-hub communities may play a crucial role in subduing pathogen colonization.

#### 3.2.4. Changes in the relative proportion of hub ASVs belonging to microbial families

In Figures 10C,D, we showed the relative proportion of hub ASVs assigned to their corresponding family levels in both the healthy and infected cohorts. From these two figures we not only observe a change in the relative proportion of hubs between the healthy and infected cohort at each time-point, but also a change across all the time-points in the infected cohort.

#### 3.2.5. Identification of dominant microbial families

**Figure 11:**
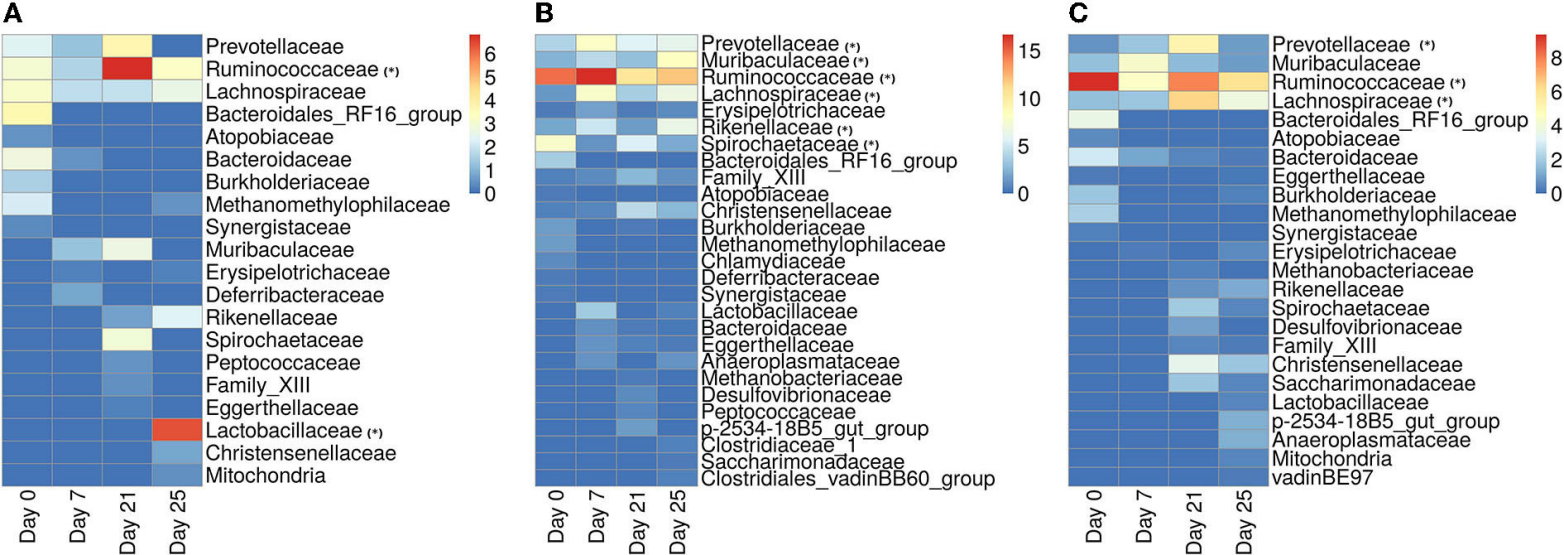
Heatmap showing the relative abundance of ASVs aggregated at the family level. **(A)** ASVs belonging to the infected-core-hub communities. **(B)** ASVs identified as hubs in the infected cohort. **(C)** ASVs identified as key-drivers in the infected cohort. Microbial families marked with “*” are identified as dominant families.

In Figure 11, we show the heatmap of the relative abundance of ASVs aggregated at their family levels and selected the microbiome families which contain ASVs having a mean-relative abundance greater than 5%. The selected microbial families are referred to as dominant families in this work and further used for unveiling their functional roles using the metaproteome data.

#### 3.2.6. ASVs belonging to a non-dominant microbial family were found as kinless hubs

In Figure 10D, it is of interest to see that ASVs assigned to the family *Desulfovibrionaceae* are found to act as kinless hubs. Although *Desulfovibrionaceae* was not found as a dominant family in our analysis at day 21, it is known to produce hydrogen sulfide (*H*_2_*S*), higher concentration of which is reported to increase the seriousness of IAV infection (Santana et al., 2021), while a reduced amount of *H*_2_*S* is found to protect against the viral infection (Dilek et al., 2020).

#### 3.2.7. Functional roles of the proteins of dominant microbial families

In order to identify the microbial functions associated with both the healthy and infected states and the corresponding protein biomarkers, we performed a Linear discriminant analysis Effect Size (LEfSe) analysis using the abundance of microbial functions obtained from our metaproteome experiment.

**Figure 12:**
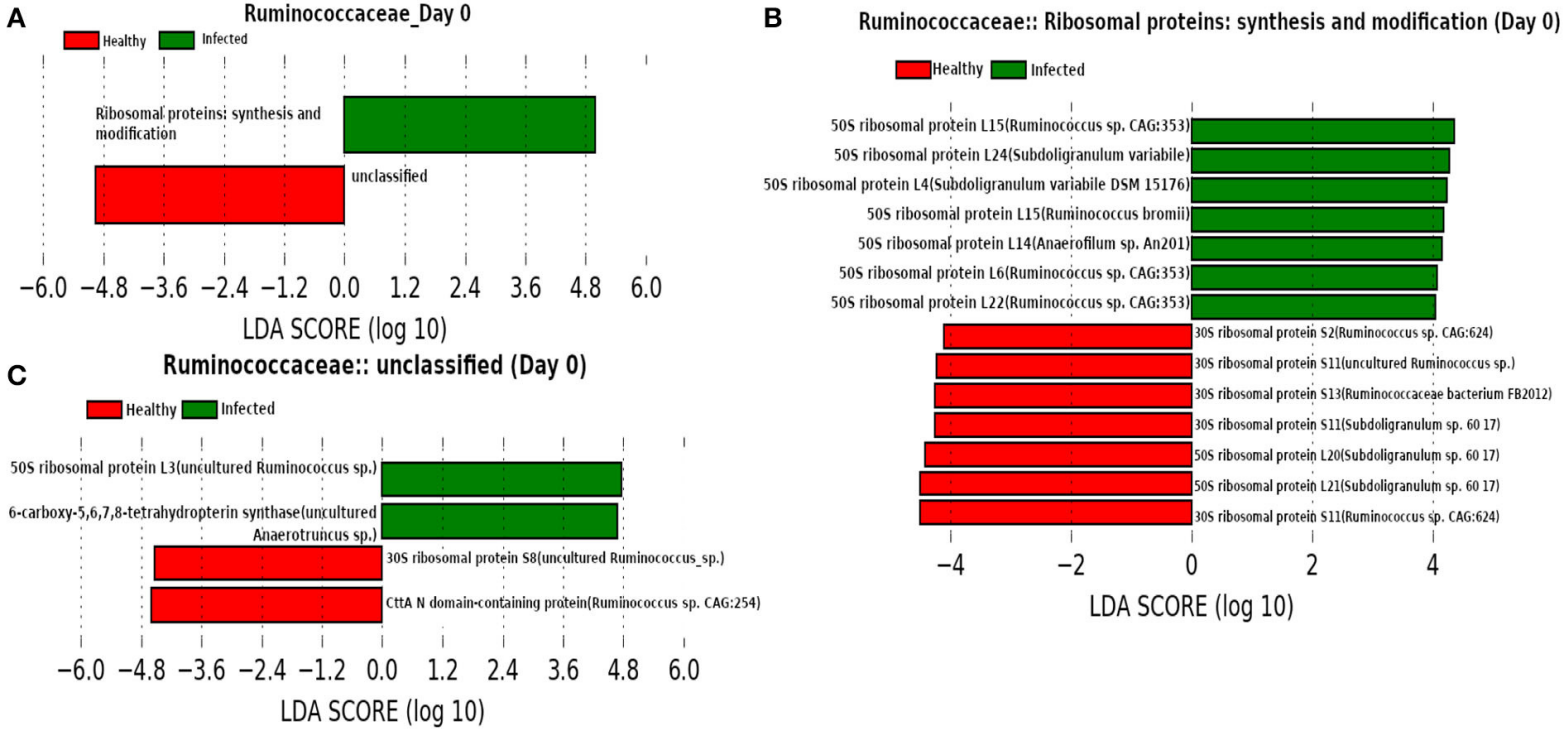
**(A)** Linear discriminant analysis effect size (LEfSe) scores of the enriched functions of the dominant microbiome family, ASVs of which are found to be the member of infected-core-hub community or act as hubs or key-drivers in the infected cohort at day 0. **(B,C)** Linear discriminant analysis effect size (LEfSe) scores of the predicted biomarkers involved in the enriched microbial functions associated with healthy and infected cohorts at day 0.

At day 0 (Figure 12) we found family *Ruminococcaceae* as a dominant family. *Ruminococcaceae* have previously been reported to be associated with H1N1-infected animals (Sencio et al., 2020). In addition, LEfSe analysis revealed the association of “Ribosomal proteins synthesis and modification” with the infected cohorts. A previous study reported that the ribosomal proteins not only play an instrumental role to trigger the viral infection by interacting with viral proteins, but also are involved in activating immune pathways against viral infection (Li, 2019).

**Figure 13:**
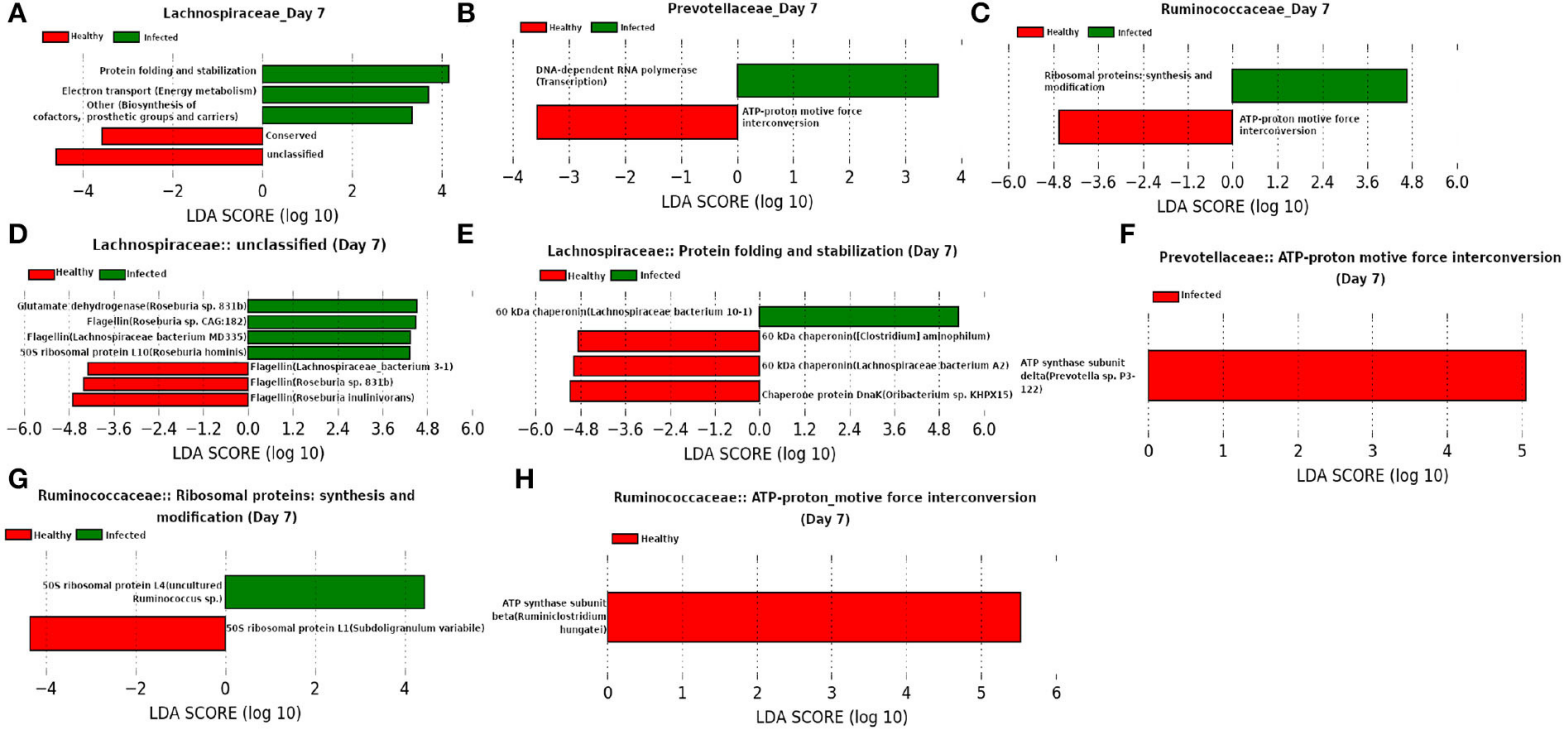
**(A–C)** Linear discriminant analysis effect size (LEfSe) scores of the enriched functions of the dominant microbiome family, ASVs of which are found to be the member of infected-core-hub community or act as hubs or key-drivers in the infected cohort at day 7. **(D–H)** Linear discriminant analysis effect size (LEfSe) scores of the predicted biomarkers involved in the enriched microbial functions associated with healthy and infected cohorts at day 7.

Besides *Ruminococcaceae*, families *Prevotellaceae* and *Lachnospiraceae* were found to be dominant at day 7 (Figure 13). Both of these families are found to be linked to immune response against Influenza A virus infection (Borey et al., 2021). LEfSe analysis of the functional composition of those microbial families revealed a lower relative abundance for “ATP-proton motive force interconversion” which may indicate the inhibition of viral replication during influenza infection by disrupting the proton motive force (PMF) (Domenech et al., 2020). It is of interest to see that our LEfSe analysis based on the relative abundance of proteins participating in the enriched microbial functions shows a higher relative-abundance of Glutamate dehydrogenase in the infected cohort because a higher level of glutamine is reported to be essential in the immune system cells during infection (de Oliveira et al., 2016). Moreover, a decrease in the relative-abundance of a well known virulence factor flagellin may be caused due to the activation of innate immune response (Hayashi et al., 2001). In addition, chaperonins are found to be prominent from our LEfSe analysis and Young (1990) showed that targeting chaperonins by the immune response plays both the protective and pathogenic roles.

**Figure 14:**
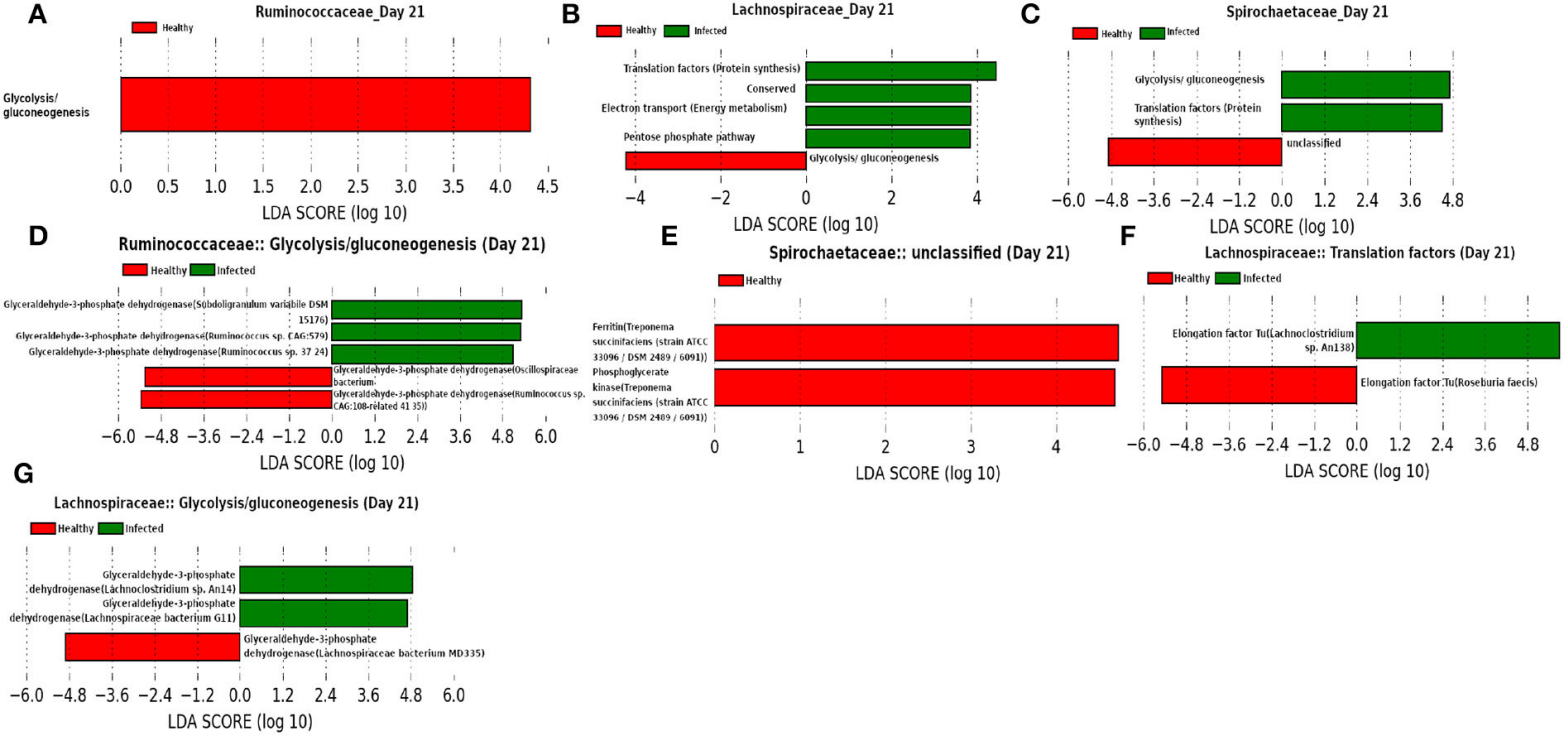
**(A–C)** Linear discriminant analysis effect size (LEfSe) scores of the enriched functions of the dominant microbiome family, ASVs of which are found to be the member of infected-core-hub community or act as hubs or key-drivers in the infected cohort at day 21. **(D–G)** Linear discriminant analysis effect size (LEfSe) scores of the predicted biomarkers involved in the enriched microbial functions associated with healthy and infected cohorts at day 21.

In addition to the *Ruminococcaceae* and *Lachnospiraceae* families, *Spirochaetaceae* is found to be a dominant family at day 21, as shown in Figure 14. Moreover, our network analysis predicted the involvement of *Ruminococcaceae* in subduing pathogen colonization. Interestingly, our prediction is supported by the findings from LEfSe analysis of the functional composition of the family *Ruminococcaceae*. We observed a lower mean relative abundance of “glycolysis” in the infected cohort suggesting a significant reduction in the viral replication (Kohio and Adamson, 2013). Furthermore, LEfSe analysis of the relative abundance of proteins belonging to the function “unclassified” revealed a lower abundance of iron storage protein ferritin in the infected cohort, indicating a recovery from infection (Lalueza et al., 2020; Perricone et al., 2020). In addition, “glycolysis” and “pentose phosphate pathway” functions of the families *Spirochaetaceae* and *Lachnospiraceae*, respectively, were found to be enriched in the infected cohorts. These findings suggest that glycolysis may play an instrumental role in the activation of the innate immune response, by rising the metabolic flux through the pentose phosphate pathway (Ganeshan and Chawla, 2014). Moreover, *Elongation factor Tu (EF-Tu)*, assigned to the function “translation factors (protein synthesis)” of the family *Lachnospiraceae* is prominent in the infected cohort. The prokaryotic EF-Tu is reported to play critical role in both the enhancement of virulence factor and activation of the host immune system (Harvey et al., 2019). Furthermore, glyceraldehyde 3-phosphate dehydrogenase (GAPDH) is found to be a marker associated with the function “glycolysis.” Sheng and Wang (2009) and Awan (2021) reported the role of GAPDH in the immune system and hence, it might be used as a therapeutic target.

**Figure 15:**
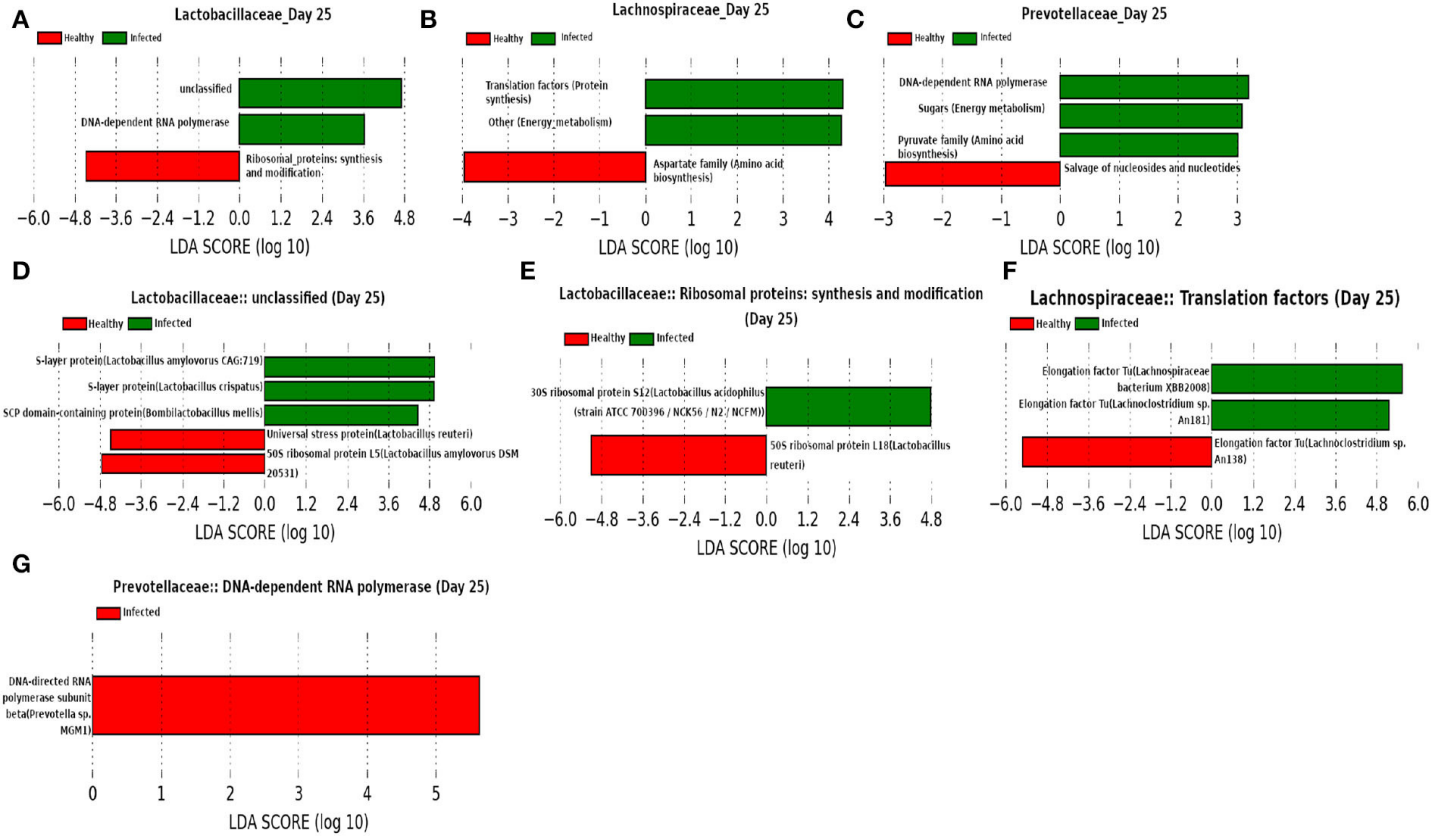
**(A–C)** Linear discriminant analysis effect size (LEfSe) scores of the enriched functions of the dominant microbiome family, ASVs of which are found to be the member of infected-core-hub community or act as hubs or key-drivers in the infected cohort at day 25. **(D–G)** Linear discriminant analysis effect size (LEfSe) scores of the predicted biomarkers involved in the enriched microbial functions associated with healthy and infected cohorts at day 25.

At day 25 (Figure 15) our network analysis based on 16srRNA data predicts the role of *Lactobacillaceae* family in subduing pathogen colonization. LEfSe analysis of relative abundance of the proteins assigned to “unclassified” function reveals the association of surface-layer proteins with the infected cohort. Acosta et al. (2019) and Wakai et al. (2021) reported that a higher concentration of the surface-layer protein of *Lactobacillaceae* family inhibits the viral replication. Since the relative abundance of S-layer protein was found to be higher in the infected cohort compared to the healthy cohort, we hypothesize that the S-layer protein might play an instrumental role in combating influenza A infection. Besides, it is of interest to see the function “pyruvate biosynthesis” of family *Prevotellaceae* and “aspartate biosynthesis” of family *Lachnospiraceae* have a relatively higher and lower composition in the infected cohort, respectively. The effect of pyruvate in alleviating influenza A virus infection is reported in Reel and Lupfer (2021), whereas aspartate is known to be crucial for viral genome nucleotide synthesis (Lao-On et al., 2018).

## 4. Discussion

In this work we proposed a maximal-clique based community detection algorithm to find modules in a weighted undirected network. We compared the performance of the proposed algorithm with some of the existing algorithms using three sets of benchmark networks and found that the proposed algorithm results in either the best or very similar performance. In addition, we applied our algorithm to a microbiome data set containing the abundance of microbial ASVs obtained from 16S rRNA gene sequencing in order to unveil the role of the gut microbiome in the host immune response during IAV infection. Our Network analysis predicts the association of microbial families such as *Ruminococcaceae, Lachnospiraceae, Spirochaetaceae, Prevotellaceae, Lactobacillaceae* with the immune response of infected cohort. In particular, we found the role of a low-abundant microbial family *Desulfovibrionaceae* as a kinless hub at day 21 and this finding may indicate its role in initiating the stabilization of gut microbial communities by producing a lower concentration of hydrogen sulfide after the second IAV infection. Moreover, the integration of metaproteome data not only provided the functions of the aforementioned microbial families in the host metabolism closely linked to immune response, but also unveiled the biomarker proteins of those dominant families. At day 25, we observed a prominent role of surface layer proteins of *Lactobacillaceae* family which may inhibit viral infection and thus, lead to fast recovery of infected pigs. Taken together, our results provided insights into the involvement of the gut microbiome and their proteins which might be beneficial for the development of novel antiviral therapy against influenza A viral infection.

## Data availability statement

16S rRNA gene sequences are available at European Nucleotide Archive (ENA), with the project number RJEB39963 (accession number ERP123542) for healthy cohort, PRJEB42450 (accession number ERP126308) for infected cohort and the project name “KoInfekt multi-omics-pipeline-swine.” Metaproteomics data is available at ProteomeXchange Consortium (submitted via the PRIDE partner repository) with the dataset identifier PXD020775.

## Ethics statement

All animal experiments were approved by the State Office for Agriculture, Food Safety and Fishery in Mecklenburg-Western Pomerania (LALFF M-V) with reference number 7221.3-1-035/17.

## Author contributions

AB developed the method, implemented the software, conducted the case studies, and drafted the manuscript. AM and HW carried out the 16s rRNA sequencing experiment. LG carried out the metaproteomics experiment. TS, CK, and CS carried out the animal case study. LK did the initial planning together with AB, discussed the results and revised the manuscript. LK, TU, KR, and TCM acquired the funding. All authors read, reviewed, and approved the final manuscript.

## Funding

This research was funded by Federal Excellence Initiative of Mecklenburg-Western Pomerania and European Social Fund (ESF) Grant KoInfekt (ESF/14-BM-A55-0014/16). LK and AB acknowledge funding from the European Union (EuCanShare, Grant No. 825903). We acknowledge support for the Article Processing Charge from the DFG and the Open Access Publication Fund of the University of Greifswald.

## Conflict of interest

The authors declare that the research was conducted in the absence of any commercial or financial relationships that could be construed as a potential conflict of interest.

## Publisher's note

All claims expressed in this article are solely those of the authors and do not necessarily represent those of their affiliated organizations, or those of the publisher, the editors and the reviewers. Any product that may be evaluated in this article, or claim that may be made by its manufacturer, is not guaranteed or endorsed by the publisher.
