# Supplementary material for: Application of a maximal-clique based community detection algorithm to gut microbiome data reveals driver microbes during influenza A virus infection
Source: Front Microbiol. 2022 Oct 20;13:979320. doi: 10.3389/fmicb.2022.979320 (PMC9630851; doi:10.3389/fmicb.2022.979320)
Supplement: Supplementary file 1 [file Data_Sheet_1.pdf]

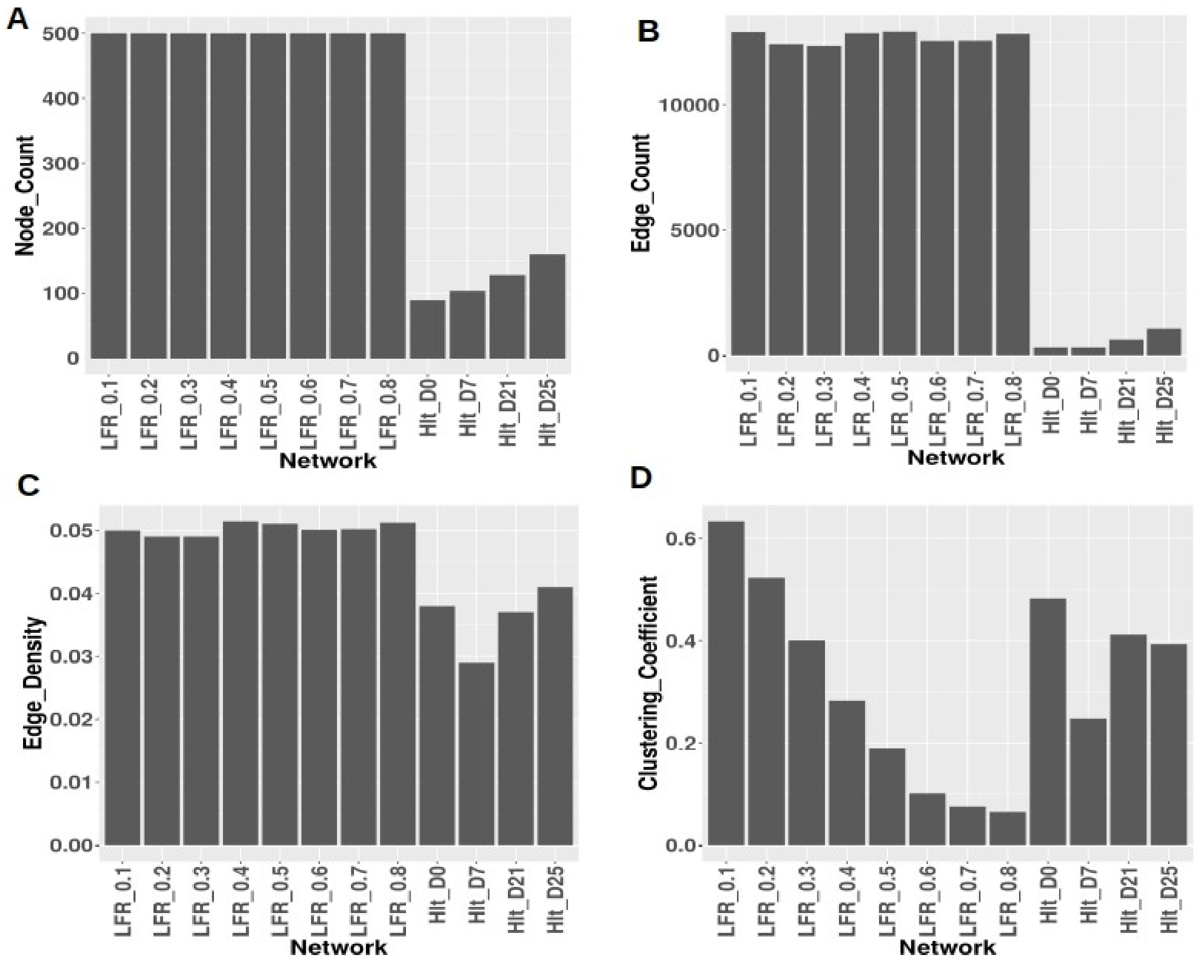

Supplementary Figure 1: Comparison between LFR benchmark networks (AN_1) and the healthy co-abundance networks in terms of **(A)** number of nodes, **(B)** number of edges, **(C)** edge density and **(D)** clustering coefficient

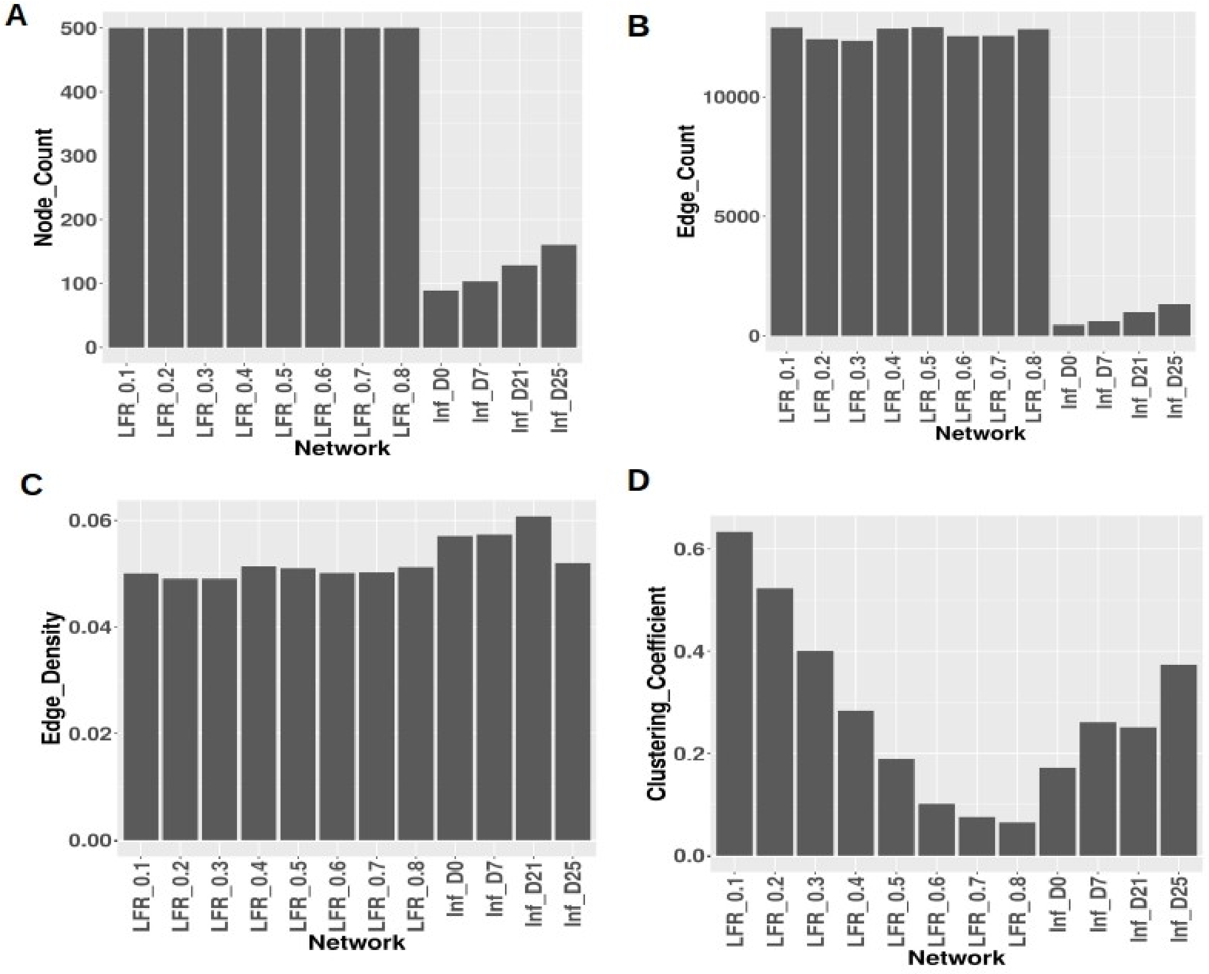

Supplementary Figure 2: Comparison between LFR benchmark networks (AN_1) and the infected co-abundance networks in terms of **(A)** number of nodes, **(B)** number of edges, **(C)** edge density and **(D)** clustering coefficient

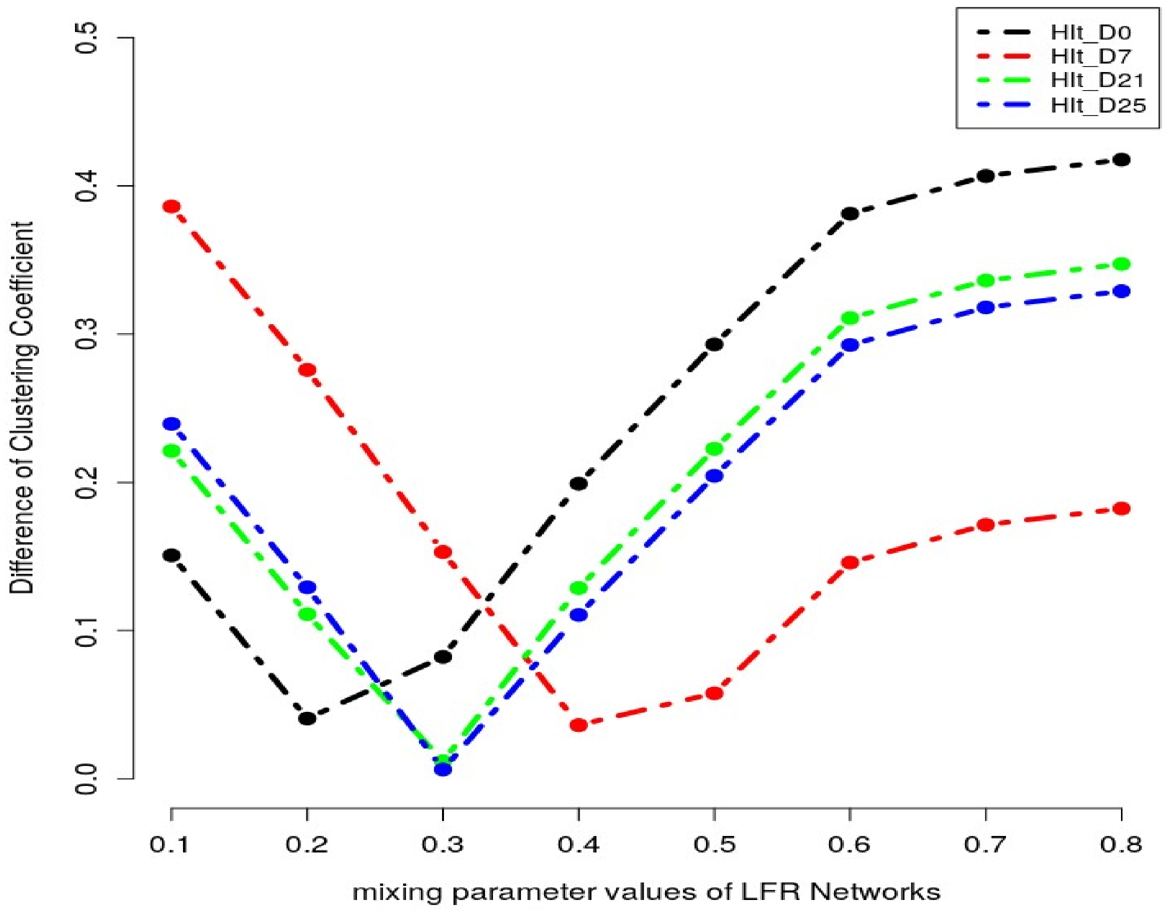

Supplementary Figure 3: Absolute difference between clustering coefficients of healthy co-abundance network and LFR benchmark networks (AN_1)

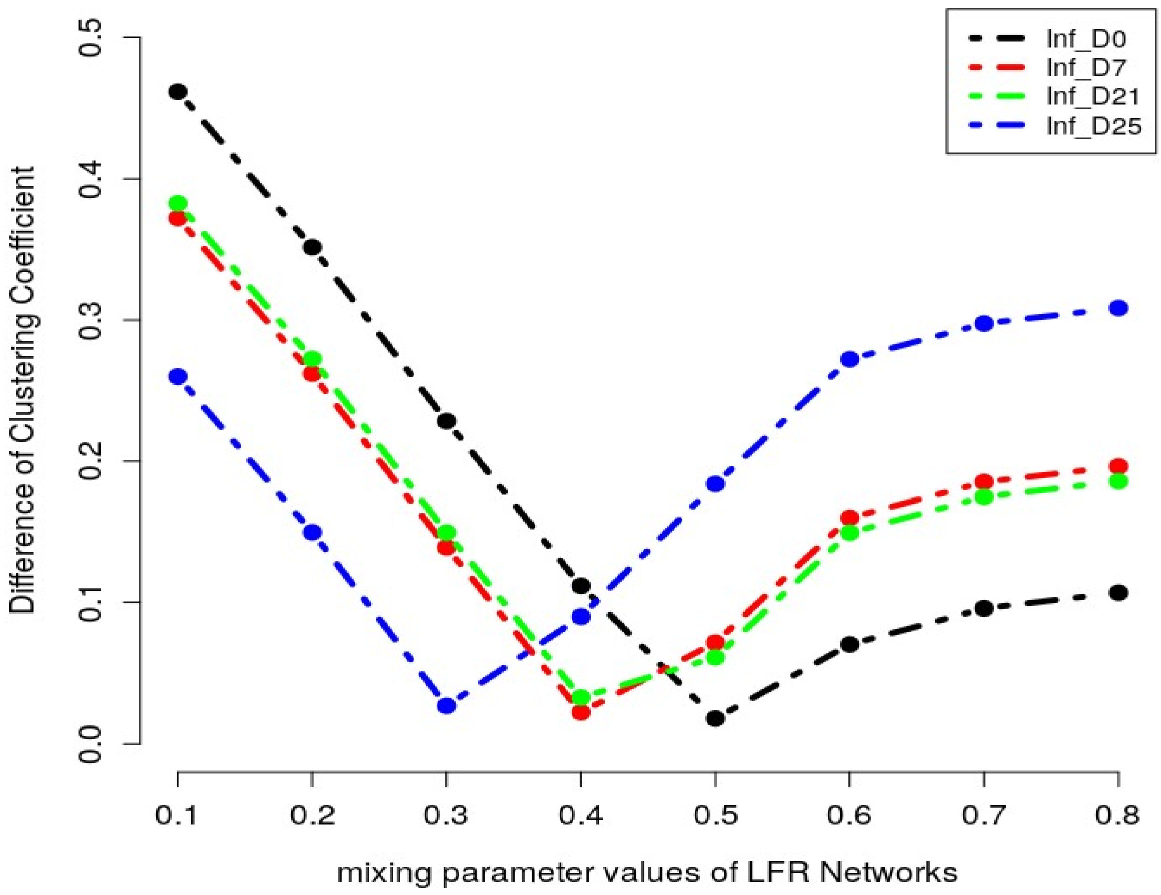

Supplementary Figure 4: Absolute difference between clustering coefficients of infected co-abundance network and LFR benchmark networks (AN_1).

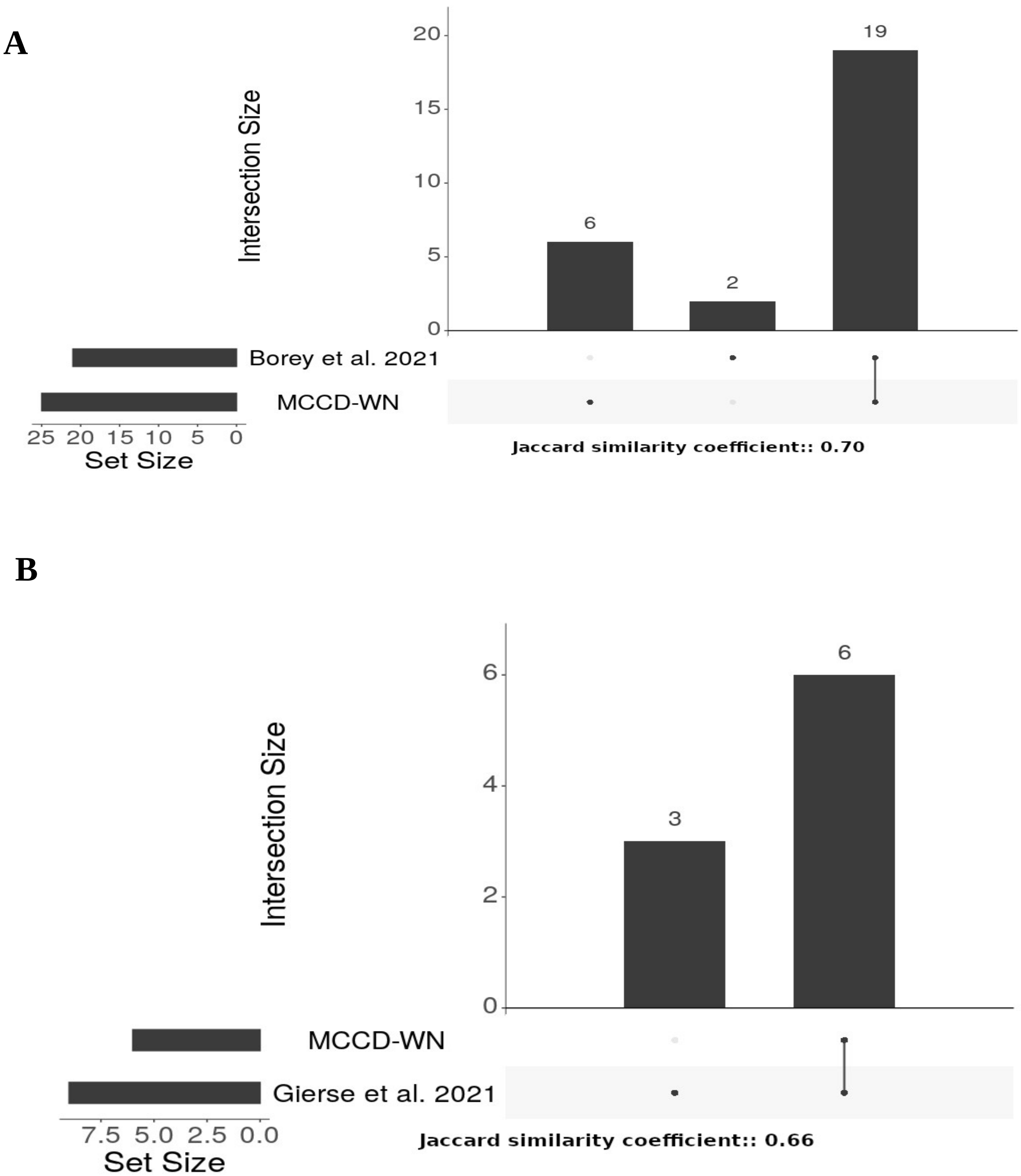

Supplementary Figure 5: Intersection between the microbiome families found by the proposed workflow and **(A)** reported to be important in Borey, M et al., 2021 and **(B)** Gierse, LC et al., 2021.
